# Public awareness, attitudes, and concerns regarding pharmacogenomics in the Kingdom of Saudi Arabia: an exploratory online cross-sectional survey

**DOI:** 10.3389/fpubh.2026.1887596

**Published:** 2026-08-24

**Authors:** Saad A. Aldosari, Dhuha Alhuthaily, Gadah K. Alonazi, Mohammed Almunef, Talal Alothman

**Affiliations:** 1 Department of Clinical Pharmacy, College of Pharmacy, Prince Sattam Bin Abdulaziz University, Alkharj, Saudi Arabia; 2 College of Pharmacy, Prince Sattam Bin Abdulaziz University, Alkharj, Saudi Arabia; 3 Pharmaceutical Care Department, Prince Sattam bin Abdulaziz University Hospital, Alkharj, Saudi Arabia; 4 Department of Pharmacy Practice, College of Pharmacy, Qassim University, Buraidah, Saudi Arabia

**Keywords:** awareness, Kingdom of Saudi Arabia, pharmacogenomics, precision medicine, public attitudes, concerns, PGX testing

## Abstract

**Background:**

Pharmacogenomics (PGx) is a cornerstone of precision medicine, enabling tailored drug therapy based on an individual’s genetic profile. However, public awareness, attitudes, and concerns regarding PGx testing in Saudi Arabia remain largely unexplored. This study aimed to evaluate these aspects among Saudi adults and to identify associated sociodemographic and health-related factors.

**Methods:**

An exploratory, cross-sectional online survey was conducted between August and October 2025 using convenience and snowball sampling. A total of 391 eligible adults (≥18 years, non-healthcare providers) completed an anonymous questionnaire adapted from previously published instruments. Multivariable logistic and ordered logistic regression analyses were performed to examine factors associated with PGx familiarity, attitudes, concerns, willingness to undergo testing, and consent preferences.

**Results:**

Attitudes toward PGx testing were strongly positive. Between 70 and 72% of respondents expressed comfort with PGx testing to predict medication efficacy, appropriate dosing, or side effects, and 68% were comfortable when it was recommended by a healthcare provider. The most prominent concerns were privacy breaches (34% high concern) and testing cost (44% high concern). While 71% expressed concern about paying out of pocket for the test, 83% indicated greater willingness if it were free or covered by insurance. Most participants (93%) expected prior notification by their provider, and 85% favored obtaining specific consent for PGx testing. In adjusted models, good/excellent self-rated health was associated with lower odds for high privacy concern (aOR 0.26, 95% CI 0.11–0.64; *p* = 0.004) and high cost concern (aOR 0.25, 0.10–0.62; *p* = 0.003). Doctoral education was associated with a more favorable overall attitude toward pharmacogenomic testing (aOR 3.92, 1.16–13.28; *p* = 0.028), and residence in Mecca (vs Riyadh) was associated with greater willingness if covered by insurance (aOR 4.24, 1.44–12.50; *p* = 0.009).

**Conclusion:**

Acceptance of and interest in PGx testing were high in this online volunteer sample, with privacy and cost as the main barriers. Robust privacy protective measures and integration into healthcare coverage could accelerate the adoption of precision medicine in Saudi Arabia. Findings should be interpreted with caution, given the convenience sampling design and limited generalizability to the broader population.

## Introduction

Pharmacogenomics (PGx), a cornerstone of precision medicine, uses an individual’s genetic profile to predict drug response, optimize drug selection and dosing, and improve tolerability, thereby enabling clinicians to achieve better clinical outcomes and enhance patient care (1–3). Nearly three decades of research support its integration into routine clinical practice, as reflected in evidence-based guidelines from expert groups, including the Clinical Pharmacogenetics Implementation Consortium (CPIC), Dutch Pharmacogenetics Working Group (DPWG), Canadian Pharmacogenomics Network for Drug Safety (CPNDS), and French National Network of Pharmacogenetics (RNPGx) (4–6).

Despite substantial advances, PGx testing remains underutilized in clinical practice (7, 8). This persistent implementation gap stems from multiple interconnected barriers, including limited clinician and patient education, inadequate integration of PGx decision support into electronic health records, inconsistent reimbursement and high costs, a lack of rapid and standardized testing platforms, ongoing ethical and privacy concerns, and evidence gaps or discordant guidelines for many gene–drug pairs (9, 10).

Given the wide range of stakeholders involved in PGx, incorporating diverse perspectives is essential when developing new healthcare systems and policies (11). This process is further influenced by social and ethical issues shaped by national, cultural, and ethnic beliefs, traditions, and values. Consequently, social acceptance of PGx can vary significantly across countries. These differences underscore the need to examine public attitudes and concerns and to develop context-specific governmental strategies (12, 13).

To promote effective adoption of PGx, it is essential to engage all stakeholders through targeted awareness and education efforts. Several studies have evaluated health professionals’ knowledge and readiness to offer PGx services, both nationally and internationally. For instance, a systematic review of over 12,430 pharmacists and pharmacy students across 26 countries found positive attitudes toward PGx but also reported inadequate knowledge, low confidence, and strong demand for training (14). Similar patterns are observed in Saudi Arabia, where clinicians and pharmacists recognize PGx benefits but cite limited knowledge, a lack of guidelines, and cost concerns (15).

While clinician-related barriers are well documented, patient and public perspectives remain underexplored yet pivotal factors that directly influence testing uptake, comprehension of results, and adherence to genotype-guided recommendations (16, 17). Large-scale surveys consistently demonstrate high levels of interest, with acceptance rates frequently exceeding 80–90% when participants are informed about potential benefits such as reduced adverse drug reactions, improved treatment efficacy, and personalized dosing (17–19). This enthusiasm is particularly pronounced among individuals with prior experience of medication side effects or greater familiarity with general genetic concepts (17, 18). However, positive attitudes do not always translate directly into action; concerns about genetic privacy, fear of insurance or employment discrimination, data security, and out-of-pocket costs remain significant barriers for many respondents, especially in underserved or historically marginalized communities (16, 17, 20).

In Saudi Arabia, research on PGx has largely focused on healthcare professionals; public attitudes and concerns regarding PGx testing remain largely unexplored. The present study aims to fill this gap by assessing these domains among a sample of Saudi adults recruited online and by identifying sociodemographic and health-related factors associated with public perception and acceptance of PGx testing.

## Materials and methods

### Study design and participants

This study was designed as an exploratory, cross-sectional online survey, conducted between August and October 2025. Eligible participants were adults aged ≥18 years residing in the Kingdom of Saudi Arabia (KSA) who provided informed consent. Healthcare providers and participants with substantially incomplete responses to the questionnaire were excluded. In the absence of a national community sampling framework, convenience and snowball sampling methods were employed; the resulting sample; therefore, represents an online volunteer sample rather than a probability-based, nationally representative cohort. The study is reported in accordance with the Strengthening the Reporting of Observational Studies in Epidemiology (STROBE) guideline and, given the open online survey design, the Checklist for Reporting Results of Internet E-Surveys (CHERRIES) (see CHERRIES reporting below).

### Survey administration and CHERRIES reporting

The questionnaire was administered as an anonymous, self-administered online survey using Google Forms, distributed openly through social media platforms (WhatsApp and Telegram) and email. No financial or other incentives were offered. The survey first page included an information sheet describing the study purpose, the voluntary nature of participation, the anticipated time burden (approximately 8–10 min), data confidentiality, the principal investigator’s contact details, and ethics approval. Electronic informed consent was required before access to the questionnaire. The questionnaire was developed and tested by the study investigators. Identifying information was not collected; the survey was hosted at a single Google Forms URL with no IP or cookie tracking enabled, and duplicate submissions could not be programmatically prevented because of the anonymous design. Duplicates were not detected through visual review of timestamps and demographic patterns. All questions were displayed on a single page with no adaptive logic; key items (consent, eligibility, sociodemographics, attitudes, and concerns) were mandatory, and participants could review and change their answers using a “Back” button prior to submission. Of 479 individuals who accessed the survey, 391 met the eligibility criteria and provided analyzable responses (analytic completion rate: 81.6%; see Results, Baseline characteristics, for full exclusions).

### Questionnaire development and measures

The questionnaire was developed from a review of the literature and previously published instruments assessing attitudes and concerns toward PGx testing and consisted of four sections: (i) sociodemographic and health-related characteristics; (ii) self-reported history of genetic testing and familiarity with PGx (preceded by a brief lay explanation of PGx testing); (iii) attitudes toward PGx testing (9 items, 5-point Likert); and (iv) concerns and uncertainties (7 items on a 3-point scale [Part A], 4 yes/no items [Part B], and 2 agree/disagree items [Part C]). Attitude items were adapted from prior public attitude surveys on PGx testing (1, 4, 21), and concern items were adapted from two previously published studies (1, 21).

### Pilot testing

The questionnaire underwent two-stage pilot testing to evaluate clarity, comprehensibility, content validity, and practicality. A preliminary pilot was conducted with 4 pharmacists to assess face validity and item relevance. A second pilot was performed with 10 individuals from the target population (non-healthcare providers). Feedback from both pilots was incorporated to improve item wording, response options, and overall feasibility of survey administration.

### Sample size

The minimum sample size was estimated using the Raosoft® sample size calculator with a 95% confidence level, a ± 5% margin of error, and a 50% assumed response distribution (22). On the basis of Saudi Arabia’s estimated mid-2024 population of 35,300,280 reported by the General Authority for Statistics (GASTAT) (23), the minimum required sample size was 385 participants.

We note that the 50% value reflects the response distribution assumption used in the sample size formula and is not an achieved response rate.

### Data analysis

Outcomes were attitudes toward PGx testing (9 Likert items), concerns regarding PGx testing (7 three-point items, 4 yes/no items, and 2 agree/disagree items), and willingness to undergo testing under specified conditions. Explanatory variables included gender, age, nationality, education, region (Riyadh / Mecca / other), private health insurance, type of usual healthcare facility, self-rated health status, chronic disease, history of any genetic testing, and self-reported familiarity with the term “pharmacogenomics” (any vs. none).

Descriptive statistics summarized all variables; categorical variables are presented as frequencies and percentages, and continuous variables as means and standard deviations. Distributions of Likert-scale and concern items are presented as stacked horizontal bar charts. For inferential analyses, multivariable logistic regression models were fitted for dichotomous outcomes — high concern (vs low/no concern) for each Part A item, binary responses for Part B (perceived discrimination, self-pay cost, willingness if free/covered), and Part C (prior notification, specific consent) items — adjusted for all the predictors listed above. Ordered logistic regression models were fitted to selected Likert attitude items and to the 5-level self-reported familiarity outcome (with familiarity excluded from its own predictor set). An overall favorable attitude was defined as a mean Likert score ≥ 4 across the nine attitude items and was modeled with logistic regression. Adjusted odds ratios (aOR) with 95% confidence intervals and two-tailed *p*-values are reported; statistical significance was set at *p* < 0.05. Some predictor categories had small cell counts (e.g., *n* = 13 for prior genetic testing; *n* = 17 for non-Saudi nationality), and a small number of cells produced unstable estimates. Model discrimination was assessed using the area under the receiver operating characteristic curve (AUC) for binary outcomes, and overall model fit using McFadden’s pseudo R^2^ for all models. Analyses were conducted in IBM SPSS Statistics (Version 31.0; IBM Corp., Armonk, NY, USA) and Python 3.12 (statsmodels 0.14).

## Results

### Baseline characteristics

A total of 479 individuals accessed the online survey. Of these, 77 were excluded because they were healthcare providers, 4 did not provide consent, and 7 were excluded for substantially incomplete responses, leaving a final analytic sample of 391 respondents who completed the core questionnaire (Table 1). Most respondents were female (289/391, 73.9%), and the mean age was 32.7 years (SD 11.3). Nearly all were Saudi nationals (374/391, 95.7%). Educational attainment was high: 259 (66.2%) held a bachelor’s degree, 43 (11.0%) a master’s or doctoral degree, 39 (10.0%) a diploma, and 50 (12.8%) a high school diploma or lower. Participants were distributed across all 13 provinces of Saudi Arabia, with almost half residing in Riyadh (176/391, 45.0%), followed by Mecca (78/391, 19.9%) and Medina (28/391, 7.2%). Only 76 (19.4%) reported having private health insurance, and most (272/391, 69.6%) usually received care from government facilities. Nearly all respondents rated their current health positively [178 (45.5%) excellent, 189 (48.3%) good]. Just over one quarter (109/391, 27.9%) reported at least one chronic disease.

**Table 1:** Sociodemographic and health-related characteristics of participants overall and by gender (*n* = 391).

Characteristic	Total number (%) (*N* = 391)	Men total number (%) (*n* = 102)	Women total number (%) (*n* = 289)
Age (years), mean (SD)	32.7 (11.3)	37.8 (12.3)	30.9 (10.3)
Age group			
18–34	246 (62.9)	48 (47.1)	198 (68.5)
35–49	101 (25.8)	32 (31.4)	69 (23.9)
> = 50	44 (11.3)	22 (21.6)	22 (7.6)
Nationality			
Saudi	374 (95.7)	92 (90.2)	282 (97.6)
Non-Saudi	17 (4.3)	10 (9.8)	7 (2.4)
Educational qualification			
High school or less	50 (12.8)	26 (25.5)	24 (8.3)
Diploma	39 (10.0)	13 (12.7)	26 (9.0)
Bachelor’s degree	259 (66.2)	39 (38.2)	220 (76.1)
Master’s degree or equivalent	18 (4.6)	5 (4.9)	13 (4.5)
Doctorate or equivalent	25 (6.4)	19 (18.6)	6 (2.1)
Province			
Riyadh	176 (45.0)	67 (65.7)	109 (37.7)
Mecca	78 (19.9)	9 (8.8)	69 (23.9)
Medina	28 (7.2)	3 (2.9)	25 (8.7)
Eastern	21 (5.4)	3 (2.9)	18 (6.2)
Asir	23 (5.9)	6 (5.9)	17 (5.9)
Tabuk	16 (4.1)	0 (0.0)	16 (5.5)
Qassim	13 (3.3)	4 (3.9)	9 (3.1)
Jazan	10 (2.6)	3 (2.9)	7 (2.4)
Najran	9 (2.3)	3 (2.9)	6 (2.1)
Northern Borders	5 (1.3)	1 (1.0)	4 (1.4)
Hail	4 (1.0)	2 (2.0)	2 (0.7)
Jawf	4 (1.0)	0 (0.0)	4 (1.4)
Bahah	3 (0.8)	1 (1.0)	2 (0.7)
Private health insurance			
Yes	76 (19.4)	21 (20.6)	55 (19.0)
No	315 (80.6)	81 (79.4)	234 (81.0)
Type of healthcare facility			
Government	272 (69.6)	81 (79.4)	191 (66.1)
Private	119 (30.4)	21 (20.6)	98 (33.9)
Self-rated health status			
Excellent	178 (45.5)	45 (44.1)	133 (46.0)
Good	189 (48.3)	50 (49.0)	139 (48.1)
Fair	22 (5.6)	5 (4.9)	17 (5.9)
Poor	2 (0.5)	2 (2.0)	0 (0.0)
Any chronic disease			
No chronic disease	282 (72.1)	65 (63.7)	217 (75.1)
≥1 chronic disease	109 (27.9)	37 (36.3)	72 (24.9)

Values are n (%) unless otherwise noted. Percentages within gender columns are computed using the respective gender total (Men n = 102, Women n = 289).

### Self-reported history of genetic testing and familiarity with PGx

A small proportion of participants had previously undergone any genetic test (13/391, 3.3%). Regarding PGx awareness, nearly half were completely unfamiliar with the term “pharmacogenomics” (177/391, 45.3%), while 105 (26.9%) were somewhat familiar, 70 (17.9%) moderately familiar, 17 (4.3%) very familiar, and 22 (5.6%) extremely familiar. Only a small minority (29/391, 7.4%) reported that a healthcare provider had ever discussed PGx concepts with them, compared with 362 (92.6%) who had not (Table 2).

**Table 2:** Self-reported history of genetic testing and familiarity with PGx (*n* = 391).

Variable	*n* (%)
Ever undergone a genetic test	
Yes	13 (3.3)
No	378 (96.7)
Familiarity with the term “pharmacogenomics”	
Completely unfamiliar	177 (45.3)
Somewhat familiar	105 (26.9)
Moderately familiar	70 (17.9)
Very familiar	17 (4.3)
Extremely familiar	22 (5.6)
Provider ever discussed pharmacogenomics concepts	
No	362 (92.6)
Yes	29 (7.4)

### Attitudes toward PGx testing

**Figure 1:**
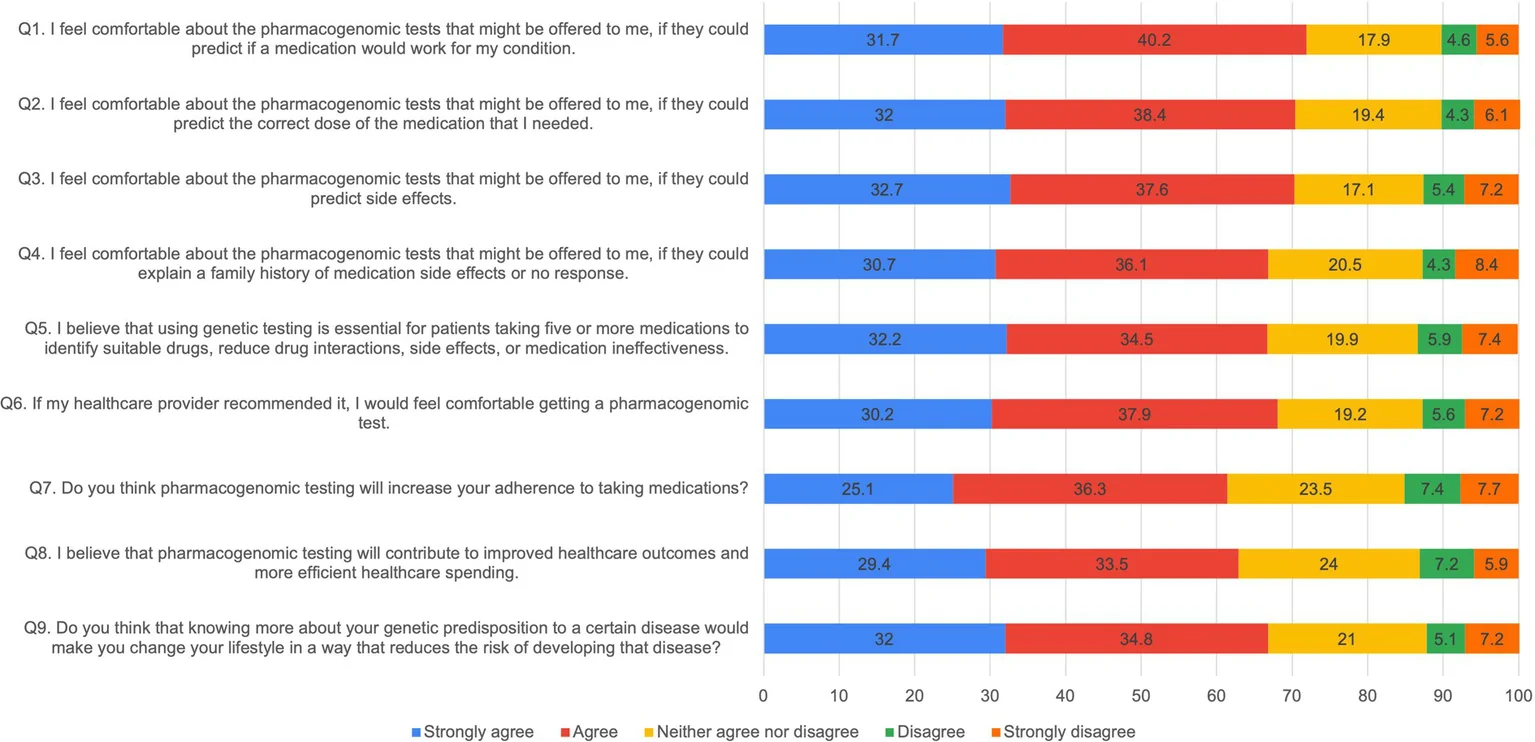
Attitudes toward pharmacogenomic testing among Saudi adults (9 items on a 5-point Likert scale: strongly disagree to strongly agree). The stacked bars show the distribution of responses, with the majority of participants expressing positive attitudes (agree or strongly agree) across all items.

Attitudes toward PGx testing were predominantly positive (Figure 1). For all tested nine items, the majority of respondents expressed favorable views.

For comfort with undergoing testing, 72% (40% agree, 32% strongly agree) reported that they would feel comfortable with a PGx test if it could predict whether a medication would work for their condition (Q1), while 6% strongly disagreed, 5% disagreed, and 18% were neutral. Similarly, 70% (38% agree, 32% strongly agree) felt comfortable if the test could predict the correct dose of a medication they needed (Q2), and 70% (38% agree, 33% strongly agree) if it could predict medication side effects (Q3).

Perceptions of the clinical value of PGx testing were also favorable. Sixty-seven percent (36% agree, 31% strongly agree) felt comfortable with tests that could explain a family history of medication side effects or lack of response (Q4). A similar proportion (67, 35% agree, 32% strongly agree) agreed that genetic testing is essential for patients taking five or more medications to help identify suitable drugs and reduce interactions, side effects, and inefficacy (Q5).

Regarding willingness to undergo PGx testing, 68% (37.9% agree, 30.2% strongly agree) indicated they would feel comfortable undergoing PGx testing if recommended by their healthcare provider (Q6); 7.2% strongly disagreed, 5.6% disagreed, and 19.2% were neutral.

Participants also perceived potential behavioral and system-level benefits. Sixty percent (36% agree, 25% strongly agree) believed that PGx testing would increase their adherence to prescribed medications (Q7), and 63% (34% agree, 29% strongly agree) agreed that such testing would improve healthcare outcomes and the efficiency of healthcare spending (Q8). Finally, 67% (35% agree, 32% strongly agree) stated that knowing more about their genetic predisposition to disease would prompt them to change their lifestyle to reduce their risk (Q9).

Overall, only about 10–15% of respondents disagreed or strongly disagreed with any attitude item, while roughly 17–25% selected neutral options, indicating a generally high level of acceptance and interest in PGx testing.

### Concerns and uncertainties regarding PGx testing

**Figure 2:**
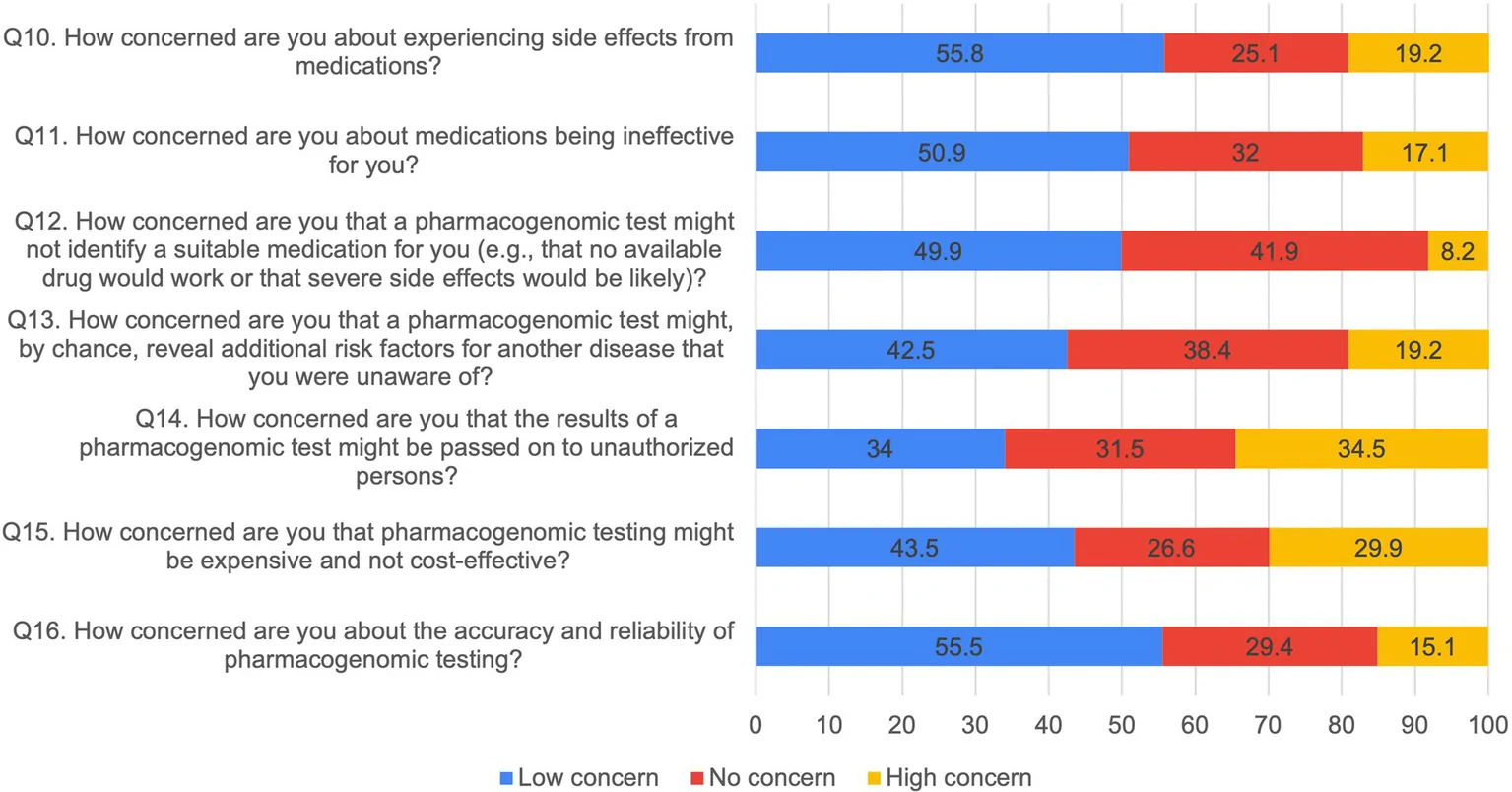
Levels of concern regarding different aspects of pharmacogenomic testing (7 items on a 3-point scale: no concern, low concern, high concern).

Figure 2 summarizes levels of concern across seven domains. For most items, low concern or no concern predominated. For example, 56% of participants reported low concern and 25% no concern about experiencing medication side effects (Q10), while 19% reported high concern. Similarly, 51% had low concern and 32% no concern about medication ineffectiveness (Q11), with 17% reporting high concern.

Concerns about the limitations of PGx testing itself were modest. Half of respondents (50%) reported low concern that a PGx test might fail to identify a suitable drug (Q12), 42% had no concern, and only 8% expressed high concern. Concerns that testing might by chance reveal additional risk factors for another disease were also limited: 43% reported low concern, 38% no concern, and 19% high concern (Q13).

In contrast, concerns about privacy and cost were more pronounced. Regarding the possibility that PGx test results might be passed to unauthorized persons (Q14), 34% had low concern, 32% no concern, and 35% high concern — the highest proportion across items. Similarly, regarding worries that PGx testing might be expensive and not cost-effective (Q15), 43.5% reported low concern, 26.6% no concern, and 29.9% high concern.

With respect to the accuracy and reliability of PGx testing (Q16), 56% of respondents reported low concern and 29% no concern, while 15% expressed high concern.

These patterns suggest that respondents were generally not highly anxious about clinical or informational consequences of testing, but a sizeable minority reported pronounced concerns about privacy and potential financial burden.

### Perceived discrimination and cost-related concerns

**Figure 3:**
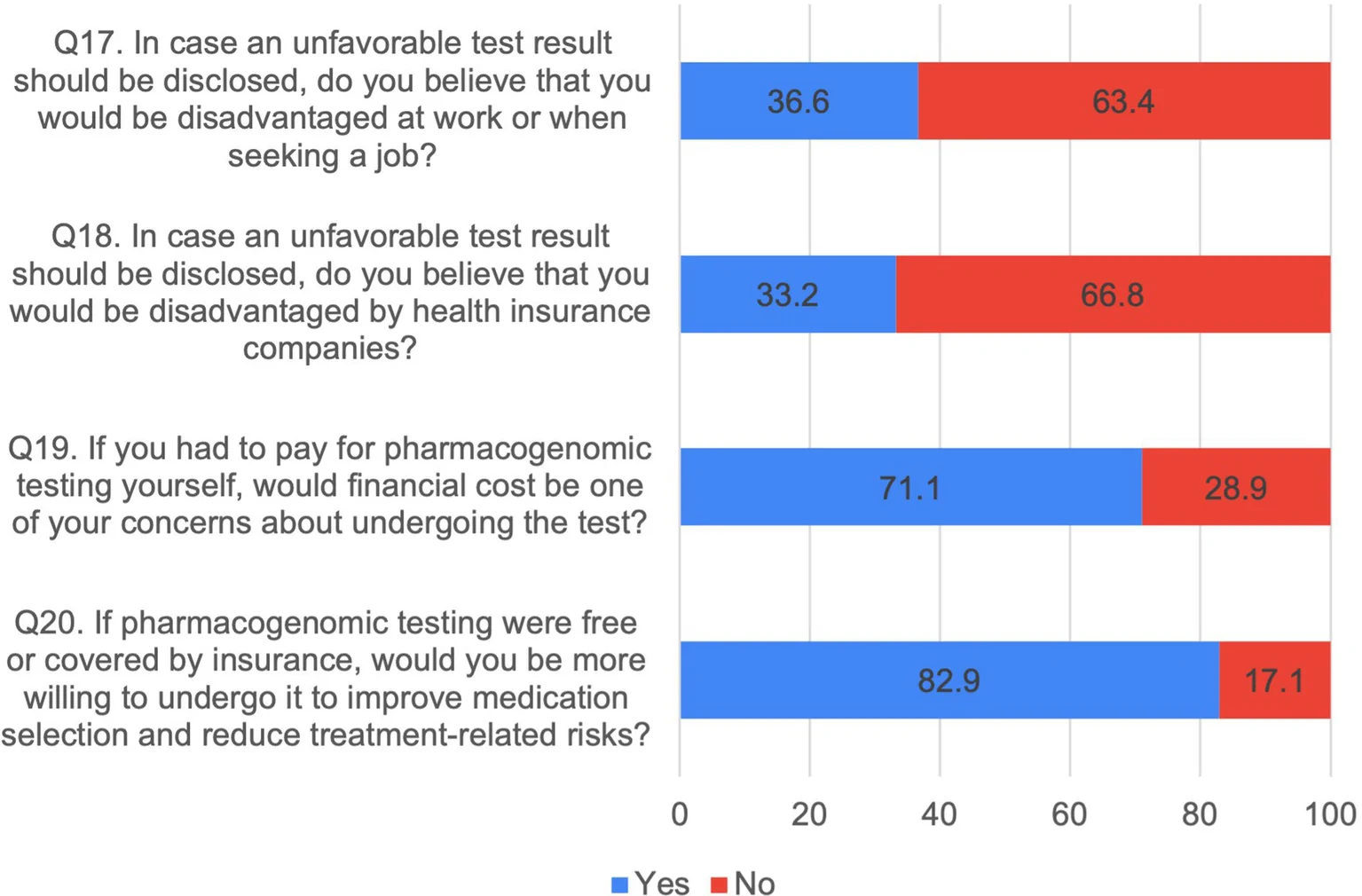
Perceived discrimination and cost-related concerns (binary yes/no items).

Binary yes/no items provided further insight into perceived discrimination and cost concerns (Figure 3). With respect to social and insurance disadvantage if an unfavorable PGx result were disclosed, 37% of participants believed they could be disadvantaged at work or when seeking employment, whereas 63% did not (Q17); likewise, 33% thought they might be disadvantaged by health insurance companies, while 67% did not expect this (Q18).

Financial issues emerged as particularly important. 71% reported that if they had to pay for PGx testing themselves, the financial cost would be a concern, compared with 29% who said cost would not be a concern (Q19). Conversely, 83% indicated that if PGx testing were free or covered by insurance, they would be more willing to undergo it to improve medication selection and reduce treatment-related risks, whereas only 17% reported that coverage would not increase their willingness to undergo it (Q20).

### Preferences for communication and consent

**Figure 4:**
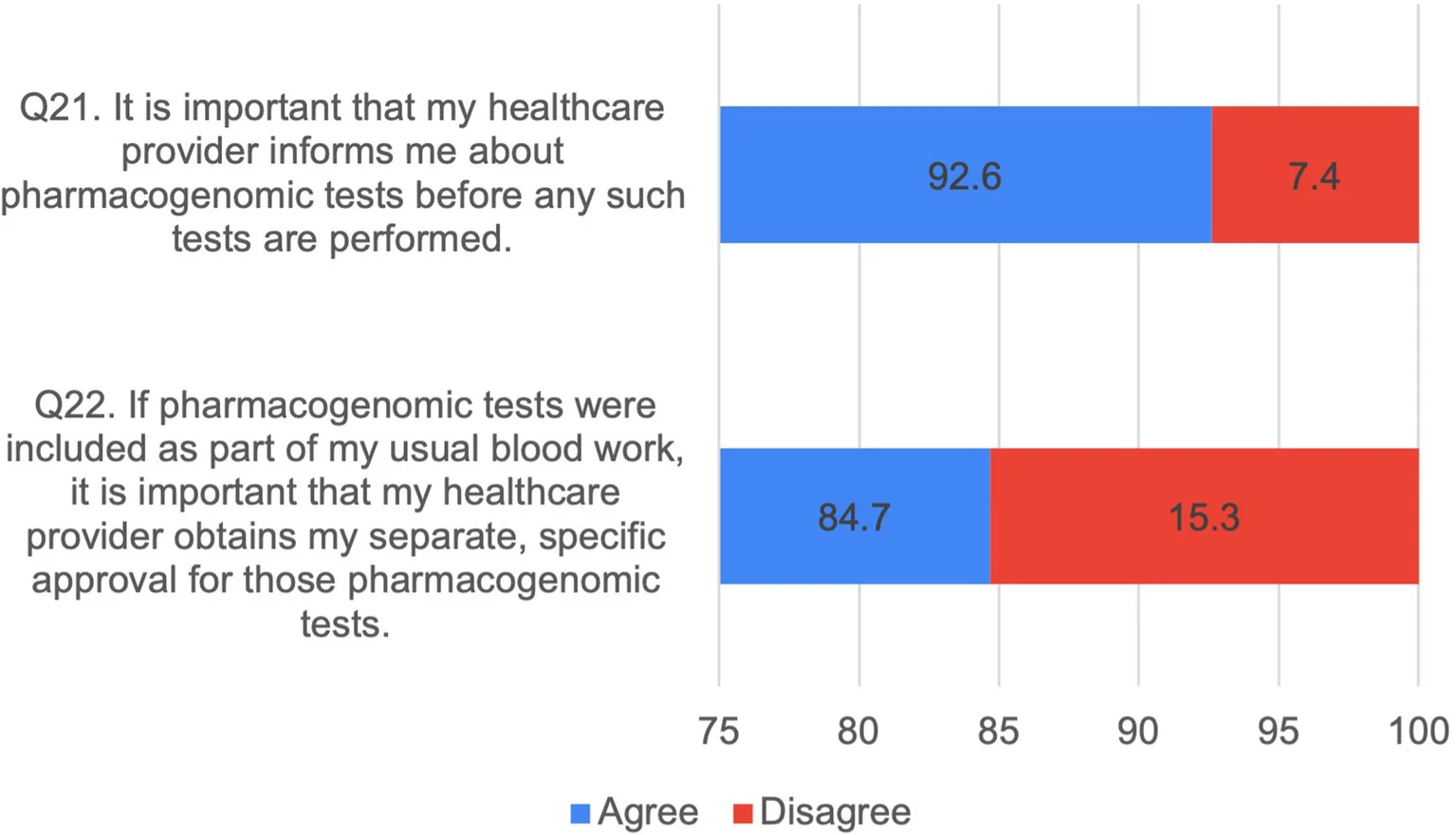
Preferences for communication and consent prior to pharmacogenomics testing (agree/disagree items).

Preferences regarding communication and consent are shown in Figure 4. Almost all participants (93%) agreed that it is important for their healthcare provider to inform them about PGx tests before any are performed, while only 7% disagreed (Q21). Similarly, 85% agreed that if PGx tests were included in their usual blood work, their healthcare provider should seek separate, specific approval for the PGx tests, whereas 15% disagreed (Q22). These responses indicate a strong expectation for transparent communication and explicit, test-specific consent when PGx testing is incorporated into routine clinical care.

### Factors associated with attitudes and concerns

Multivariable regression models were used to identify sociodemographic and health-related factors associated with high concerns, willingness to test, and an overall favorable attitude. Table 3 presents adjusted odds ratios (aOR) with 95% confidence intervals for four key outcomes: high concern about privacy (Q14), high concern about cost (Q15), increased willingness if the test were free or covered by insurance (Q20), and an overall favorable attitude (defined as a mean Likert score ≥ 4 across the nine attitude items). Models were adjusted for gender, age, nationality, education, region, private health insurance, type of usual healthcare facility, self-rated health status, chronic disease, prior genetic testing, and any familiarity with PGx.

**Table 3:** Multivariable regression models for high concerns, willingness, and overall favorable attitude (*n* = 391).

Predictor	High concern: privacy (Q14)	High concern: cost (Q15)	Willing if free/covered (Q20)	Favorable overall attitude
Female (vs male)	0.97 (0.55–1.69), *p* = 0.903	1.00 (0.56–1.79), *p* = 0.995	1.05 (0.51–2.17), *p* = 0.887	0.93 (0.54–1.58), *p* = 0.776
Age (per 1 year)	0.98 (0.95–1.00), *p* = 0.086	0.99 (0.96–1.02), *p* = 0.439	1.02 (0.99–1.06), *p* = 0.193	1.00 (0.98–1.03), *p* = 0.905
Saudi nationality (vs non-Saudi)	0.38 (0.11–1.24), *p* = 0.109	0.53 (0.16–1.70), *p* = 0.286	8.19 (1.03–65.00), p = 0.047	2.85 (0.84–9.66), *p* = 0.092
High school or less (vs Bachelor)	1.73 (0.87–3.44), *p* = 0.120	1.38 (0.66–2.87), *p* = 0.387	1.15 (0.48–2.75), *p* = 0.749	1.91 (0.96–3.80), *p* = 0.067
Diploma (vs Bachelor)	1.32 (0.60–2.89), *p* = 0.495	1.04 (0.44–2.44), *p* = 0.926	1.21 (0.45–3.29), *p* = 0.702	1.37 (0.65–2.88), *p* = 0.405
Master (vs Bachelor)	0.95 (0.32–2.82), *p* = 0.922	1.65 (0.56–4.88), *p* = 0.365	— (sparse)	2.30 (0.73–7.27), *p* = 0.157
Doctorate (vs Bachelor)	2.01 (0.66–6.12), *p* = 0.217	1.59 (0.50–5.04), *p* = 0.432	12.89 (0.89–185.77), *p* = 0.060	3.92 (1.16–13.28), p = 0.028
Mecca region (vs Riyadh)	1.18 (0.64–2.19), *p* = 0.591	0.93 (0.48–1.77), *p* = 0.817	4.24 (1.44–12.50), p = 0.009	1.77 (0.96–3.27), *p* = 0.066
Other regions (vs Riyadh)	1.00 (0.59–1.71), *p* = 0.989	0.96 (0.55–1.67), *p* = 0.892	1.00 (0.54–1.86), *p* = 0.998	0.93 (0.57–1.54), *p* = 0.789
Private insurance (vs no)	1.06 (0.57–1.98), *p* = 0.846	1.28 (0.68–2.41), *p* = 0.447	1.04 (0.45–2.39), *p* = 0.934	0.77 (0.42–1.42), p = 0.405
Private facility (vs government)	1.61 (0.92–2.82), *p* = 0.094	1.48 (0.83–2.64), *p* = 0.184	0.90 (0.44–1.86), *p* = 0.781	1.15 (0.67–1.99), *p* = 0.613
Self-rated good/excellent (vs fair/poor)	0.26 (0.11–0.64), p = 0.004	0.25 (0.10–0.62), p = 0.003	0.71 (0.19–2.67), *p* = 0.617	0.62 (0.25–1.54), *p* = 0.301
Chronic disease (vs none)	1.17 (0.68–2.00), *p* = 0.567	0.78 (0.44–1.38), *p* = 0.386	2.24 (0.99–5.09), *p* = 0.054	1.31 (0.78–2.20), *p* = 0.305
Prior genetic test (vs no)	0.55 (0.14–2.18), *p* = 0.396	— (sparse)	0.88 (0.17–4.61), *p* = 0.885	2.15 (0.55–8.42), *p* = 0.274
Any PGx familiarity (vs none)	1.32 (0.84–2.06), *p* = 0.227	1.01 (0.63–1.60), *p* = 0.975	0.92 (0.52–1.63), *p* = 0.769	0.93 (0.61–1.42), *p* = 0.735
Model fit (AUC; McFadden pseudo R^2^)	0.66; 0.053	0.65; 0.063	0.69; 0.103	0.64; 0.040

Adjusted odds ratios (aOR) with 95% confidence intervals are presented. aOR, adjusted odds ratio; CI, confidence interval. Models were adjusted for all predictors listed in the table. “— (sparse)” indicates unstable estimates due to small cell counts. The “favorable overall attitude” outcome is a logistic regression on a binary indicator of mean Likert ≥ 4 across the 9 attitude items; the Q11 column models the “Yes” response to “More willing to undergo testing if free or covered.” AUCs (binary outcomes): Privacy 0.66, cost 0.65, willingness 0.69, favorable attitude 0.64. McFadden’s pseudo R^2^: Privacy 0.053, cost 0.063, willingness 0.103, favorable attitude 0.040.

Self-rated good or excellent health (vs fair/poor) was independently associated with substantially lower odds of high concern about privacy (aOR 0.26, 95% CI 0.11–0.64; *p* = 0.004) and about cost (aOR 0.25, 0.10–0.62; *p* = 0.003). Exploratory models for side effect and ineffectiveness concerns showed that residence in Mecca (vs Riyadh) was associated with higher odds of high concern about medication side effects (aOR 3.62, 1.72–7.65; *p* = 0.001) and medication ineffectiveness (aOR 3.01, 1.42–6.35; p = 0.004), and chronic disease was associated with greater concern about ineffectiveness (aOR 2.08, 1.10–3.92; *p* = 0.024).

For willingness to undergo testing if free or covered by insurance (Q20), residence in Mecca was associated with markedly higher odds of willingness (aOR 4.24, 1.44–12.50; *p* = 0.009).

Saudi nationality also showed a positive association (aOR 8.19, 95% CI 1.03–65.00; *p* = 0.047); however, this result should be interpreted with caution due to the small number of non-Saudi respondents (*n* = 17) and the resulting wide confidence interval. For an overall favorable attitude, holding a doctoral degree (vs bachelor’s) was independently associated with higher odds of agreement (aOR 3.92, 1.16–13.28; *p* = 0.028); ordered logistic models for individual attitude items showed a similar pattern, with doctoral education associated with more positive responses on Q1 (aOR 3.24, 1.18–8.93; *p* = 0.023) and Q6 (provider-recommended testing) (aOR 2.92, 1.09–7.79; *p* = 0.033), and female gender associated with somewhat less positive responses on Q1 (aOR 0.60, 0.37–0.98; *p* = 0.040). Prior genetic testing and any prior familiarity with the term “pharmacogenomics” were not independently associated with the primary outcomes after adjustment.

Beyond the four primary outcomes shown in Table 3, we also fitted multivariable models for self-reported familiarity with PGx (5-level ordered logistic outcome, with familiarity excluded from its own predictor set), preferences for prior provider notification (Q21) and specific consent (Q22), and the remaining concern items (Q10–Q13, Q16–Q19). Adjusted estimates for familiarity and consent preferences are presented in Table 4; coefficient-level output for all models is provided in Appendix A. Younger age (aOR 0.97 per year, 95% CI 0.95–0.99; *p* = 0.009) and a prior history of any genetic testing (aOR 3.77, 1.40–10.17; *p* = 0.009) were independently associated with greater familiarity with the term “pharmacogenomics”; no other predictor reached significance. For specific consent (Q22), no predictor was independently associated after adjustment, consistent with the very high overall agreement (84.7%). For prior-notification preference (Q21), the very high agreement (92.6%) produced unstable estimates in several predictor cells (notably master’s degree, doctorate, and self-rated good/excellent health), and the corresponding estimates are unstable and reported as “— (sparse)” in Table 4; no individually significant association was identified.

**Table 4:** Multivariable regression models for self-reported familiarity with PGx and consent preferences (*n* = 391).

Predictor	PGx familiarity (5-level)	Prior notification agreed (Q21)	Specific consent agreed (Q22)
Female (vs male)	0.84 (0.51–1.36), *p* = 0.467	0.34 (0.09–1.35), *p* = 0.125	0.82 (0.40–1.70), *p* = 0.599
Age (per 1 year)	0.97 (0.95–0.99), p = 0.009	1.00 (0.95–1.05), *p* = 0.913	1.03 (0.99–1.06), *p* = 0.101
Saudi nationality (vs non-Saudi)	0.45 (0.16–1.24), *p* = 0.123	6.22 (0.39–99.77), *p* = 0.197	1.81 (0.37–8.90), *p* = 0.466
High school or less (vs Bachelor)	1.47 (0.81–2.69), *p* = 0.204	0.74 (0.22–2.49), *p* = 0.631	0.53 (0.24–1.21), *p* = 0.132
Diploma (vs Bachelor)	1.45 (0.75–2.80), *p* = 0.273	1.41 (0.29–6.94), *p* = 0.673	0.61 (0.23–1.57), *p* = 0.303
Master (vs Bachelor)	1.97 (0.81–4.79), *p* = 0.134	— (sparse)	0.87 (0.18–4.32), *p* = 0.865
Doctorate (vs Bachelor)	1.58 (0.59–4.19), *p* = 0.360	— (sparse)	0.91 (0.17–4.75), *p* = 0.908
Mecca region (vs Riyadh)	1.16 (0.67–1.98), *p* = 0.598	1.39 (0.34–5.77), *p* = 0.650	2.51 (0.94–6.71), *p* = 0.065
Other regions (vs Riyadh)	0.97 (0.62–1.53), p = 0.905	0.43 (0.17–1.10), *p* = 0.079	0.90 (0.47–1.71), *p* = 0.742
Private insurance (vs no)	1.58 (0.90–2.76), *p* = 0.111	0.69 (0.18–2.57), *p* = 0.578	1.52 (0.62–3.74), p = 0.360
Private facility (vs government)	0.74 (0.45–1.23), *p* = 0.246	3.16 (0.87–11.43), *p* = 0.080	0.90 (0.43–1.92), *p* = 0.793
Self-rated good/excellent (vs fair/poor)	0.81 (0.36–1.80), *p* = 0.604	— (sparse)	0.90 (0.24–3.30), *p* = 0.872
Chronic disease (vs none)	0.70 (0.45–1.11), p = 0.134	1.25 (0.39–3.95), *p* = 0.707	0.67 (0.33–1.33), *p* = 0.253
Prior genetic test (vs no)	3.77 (1.40–10.17), p = 0.009	0.60 (0.06–5.84), *p* = 0.662	2.32 (0.28–19.30), *p* = 0.437
Any PGx familiarity (vs none)	— (outcome)	1.84 (0.80–4.26), *p* = 0.153	1.02 (0.57–1.81), *p* = 0.954
Model fit (AUC; McFadden pseudo R^2^)	—; 0.028	0.78; 0.141	0.66; 0.043

Adjusted odds ratios (aOR) with 95% confidence intervals are presented. aOR, adjusted odds ratio; CI, confidence interval. The familiarity outcome (5-level: Completely unfamiliar to extremely familiar) was modeled with ordered logistic regression; predictors were identical to Table 1, except that “any PGx familiarity” was excluded because it is the outcome (marked “n/a”). The Q12 and Q13 outcomes are binary (agree vs. disagree). The Q12 (prior notification, 92.6% agree) model exhibits unstable estimates across multiple cells due to the very high level of agreement; “— (sparse)” entries denote these unstable estimates. AUCs (binary outcomes): Q12 0.78, Q13 0.66. McFadden’s pseudo *R*^2^: Familiarity 0.028, Q12 0.141, Q13 0.043.

Among the remaining concern outcomes, residence in Mecca (vs Riyadh) was associated with markedly higher odds of high concern about medication side effects (Q10; aOR 3.62, 1.72–7.65; *p* = 0.001) and medication ineffectiveness (Q11; aOR 3.01, 1.42–6.35; *p* = 0.004) and, conversely, lower odds of high concern about incidental findings (Q13; aOR 0.45, 0.21–0.95; *p* = 0.037). Self-rated good/excellent health was protective against high concern across Q10, Q11, and Q18 (aOR 0.26–0.40, all *p* < 0.05). Private insurance was independently associated with higher odds of concern about test limitations (Q12; aOR 3.67, 1.35–9.96; *p* = 0.011), and older age with higher odds of perceived health-insurance disadvantage (Q18; aOR 1.04 per year, 1.01–1.06; *p* = 0.004). Lower-education categories were associated with greater self-pay cost concern (Q19), with master’s-degree respondents showing a very wide confidence interval (aOR 8.20, 1.01–66.32) due to small cell size. McFadden’s pseudo-R^2^ for the binary outcomes ranged from 0.03 to 0.14, indicating that, in aggregate, the measured demographic and health-related variables account for a modest share of the variation in attitudes and concerns. Unmeasured factors, including specific prior experiences, information sources, and trust in the healthcare system, likely play substantial roles.

## Discussion

To the best of our knowledge, this is the first study in Saudi Arabia to examine public attitudes and concerns regarding PGx among a general adult population. Prior Saudi work has focused mainly on healthcare providers (14, 15); our findings provide novel context-specific evidence on how Saudi adults outside the health professions view PGx testing. Three results stand out: awareness of and prior exposure to PGx are low; attitudes are strongly positive once PGx is briefly explained; and concerns are targeted rather than diffuse, concentrating on privacy and out-of-pocket cost.

Overall, familiarity with the term “pharmacogenomics” was low: 45.3% were completely unfamiliar, 26.9% somewhat familiar, 17.9% moderately familiar, and only 9.9% very or extremely familiar. This distribution aligns with international surveys of patients and the general public. For example, Gawronski et al. reported that only 21.1% of medically underserved U. S. patients had previously heard of PGx testing before being given an explanation (24). A U. S. study by Haga et al. found high general awareness of genetic testing (80%), but very low reported experience with PGx testing (1–4%) (17), and Edris et al. (2022) reported that only 30.8% of the public in Flanders, Belgium, had heard of PGx (25). In Korea, Lee et al. found that approximately two-thirds (66%) of the general public were aware of personalized medicine in a broad sense, but only 28% understood that genetic factors contribute to inter-individual variation in drug response (26). The 45.3% rate of complete unfamiliarity in our sample is somewhat lower than some international estimates, but it still indicates limited baseline exposure; this is notable given the younger and more highly educated profile of our online sample (66.2% with a bachelor’s degree or higher, nearly half in Riyadh), characteristics typically associated with higher PGx awareness in prior studies (17, 18, 27). Limited familiarity is consistent with the very low prior exposure observed (3.3% had undergone any genetic testing, and 7.4% had discussed PGx with a provider).

Despite low familiarity, attitudes toward PGx testing were strongly positive, with 70 and 72% expressing comfort or agreement across all nine items. This high level of acceptance — particularly for predictive testing (70–72% comfort with efficacy, dose, and side-effect prediction) and provider-recommended testing (68%) — is in line with international surveys reporting 80–90% acceptance when potential benefits are explained (4, 17, 18). Perceived advantages, including improved adherence (61%), healthcare efficiency (63%), and lifestyle modification (67%), further reflect optimism about PGx as a tool for personalized care. Low disagreement rates (10–15% across items) and moderate neutral responses (17–24%) suggest broad receptivity even among a population with limited familiarity, consistent with evidence that brief educational interventions can substantially increase willingness to undergo PGx testing (4, 28).

Concerns were selective but meaningful. Clinical risks (side effects, ineffectiveness, incidental findings) elicited low concern in the majority, while privacy (35% high concern) and cost (30% high concern) were the most prominent barriers. These findings align with the global literature, which consistently identifies privacy, discrimination, and financial burden as key barriers, especially in diverse or resource-constrained settings (16, 17, 20). In Saudi Arabia, where government facilities dominate care, out-of-pocket costs may be perceived as particularly burdensome. The yes/no items reinforced this: 71% cited cost as a concern if self-paid, yet 83% indicated greater willingness if the test were covered by insurance or free of charge. Likewise, 33–37% anticipated potential insurance or employment disadvantage from an unfavorable result, reflecting ongoing concerns about genetic discrimination that persist despite legal frameworks in some regions.

The multivariable analyses provide additional insights. Self-rated good or excellent health was strongly and independently associated with lower odds of high concern about both privacy and cost (aOR ~ 0.25 for both). One interpretation is that respondents with poorer self-rated health, who are more likely to be regular users of the healthcare system, anticipate the practical consequences of genetic test results and associated costs more clearly than healthier respondents. Residence in Mecca (vs Riyadh) was consistently associated with higher concern about medication-related risks and with greater willingness to undergo testing if it were free or covered; this combination suggests a population that is attentive to medication-related risks but particularly sensitive to cost-related access barriers. Doctoral education was independently associated with a more favorable overall attitude and with more positive responses to individual attitude items, consistent with international evidence linking higher education to greater PGx acceptance (17, 18). In contrast, prior genetic testing and self-reported familiarity with the term “pharmacogenomics” were not independently associated with the primary outcomes after adjustment; with only 13 previously tested participants, however, statistical power to detect such effects was very limited. For the secondary outcomes (Table 4), younger age and prior experience with genetic testing were independently associated with greater familiarity with PGx, consistent with greater exposure to digital health information among younger Saudis and with the experiential learning that follows genetic testing. Consent preferences showed very high agreement — 92.6% agreed that providers should give prior notification (Q21) and 84.7% favored specific consent for PGx tests bundled with routine blood work (Q22). No demographic or health-related characteristic was independently associated with these preferences after adjustment, suggesting that the expectation of informed-consent safeguards is broadly shared in this sample.

Similar positive attitudes have been reported in neighboring Gulf countries. A multi-ethnic study from the United Arab Emirates found generally favorable knowledge and attitudes toward genomic medicine and genetic testing, with about three-quarters (76.9%) of participants willing to undergo testing if a relative had a genetic disease. While few considered genetic tests painful (3.4%), most viewed them as costly (64.9%) and time-consuming (59.5%) (29). Similarly, a survey in Qatar showed that approximately 71% of respondents were willing to participate in the Qatar Genome Program. Willingness was significantly associated with genetic literacy, family history of genetic diseases, and prior experience with premarital genetic screening. The main motivation was to learn more about their health, while lack of time and information were the primary barriers. These regional findings align closely with our results, in which 70–72% of Saudi participants expressed comfort with pharmacogenomic testing to predict medication efficacy, dosing, or side effects (30).

Several limitations should be considered when interpreting these findings. First, recruitment relied on convenience and snowball sampling through social media and email; the resulting sample was predominantly female, young, highly educated, and concentrated in Riyadh and Mecca, and the results are therefore best understood as applying to this online volunteer sample rather than to the Saudi adult population at large. We have framed the study accordingly as an exploratory online cross-sectional survey. Second, although the questionnaire was piloted for clarity and face validity, the adapted attitude and concern items were not formally revalidated in this sample, and no estimate of internal consistency is reported. Third, several predictor categories contained small cell counts (non-Saudi nationality, master’s and doctoral degrees, prior genetic testing), which produced wide confidence intervals and, in a small number of cases, unstable estimates; these have been flagged in Tables 3, 4 and should be interpreted with caution. In particular, the very high agreement with prior-notification preference (Q21, 93%) produced unstable estimates in several predictor cells in that model. Fourth, McFadden’s pseudo R^2^ values were modest (0.03–0.14 across the binary outcomes), indicating that the measured demographic and health-related variables account for only a small share of the variation in attitudes and concerns; unmeasured factors such as specific prior experiences and trust in the healthcare system are likely to play important additional roles. Finally, although we report multivariable models for the primary and secondary outcomes, we have not adjusted for multiple comparisons across the full set of items, in keeping with the exploratory framing of the analysis; individual associations should be considered hypothesis-generating.

These findings carry important implications for PGx implementation in Saudi Arabia. The high acceptance observed despite low familiarity suggests that public education campaigns and brief point-of-care interventions could substantially accelerate uptake, particularly if they explicitly address privacy safeguards and clarify cost coverage. Collaboration among the Ministry of Health, academic institutions, and the Saudi Food and Drug Authority could develop accessible educational materials that leverage digital platforms, in line with the strong social media engagement of our sample. Future probability-based studies with representative sampling — including older adults, rural residents, and lower-literacy groups — together with longitudinal follow-up of educational interventions, will be needed to confirm and extend these findings.

## Conclusion

In this exploratory online sample of Saudi adults, attitudes toward PGx testing were strongly positive, with high acceptance, despite limited prior familiarity with the field. Concerns were targeted rather than diffuse, centered on privacy and out-of-pocket costs, and patterned by self-rated health, region, and education. Robust privacy protective measures, transparent provider communication, specific consent processes, and the integration of PGx testing into existing healthcare coverage would address the most prominent barriers identified and could materially accelerate the equitable adoption of precision medicine in the Kingdom of Saudi Arabia.

## Data Availability

The original contributions presented in the study are included in the article/Supplementary material, further inquiries can be directed to the corresponding author.
