# Supplementary material for: Public awareness, attitudes, and concerns regarding pharmacogenomics in the Kingdom of Saudi Arabia: an exploratory online cross-sectional survey
Source: Front Public Health. 2026 Aug 24;14:1887596. doi: 10.3389/fpubh.2026.1887596 (PMC13547504; doi:10.3389/fpubh.2026.1887596)
Supplement: Supplementary file 1 [file Table_1.docx]

**Appendix A. Full multivariable regression results**

Complete coefficient-level output for the four primary models (high privacy concern, high cost concern, willingness if free/covered, and overall favorable attitude) and three secondary ordered-logistic models (attitude Q1, Q4, Q15; side-effect and ineffectiveness concern models). All values reflect maximum-likelihood estimation in statsmodels 0.14. "— (sparse)" indicates the predictor was dropped or near-separated due to zero or near-zero cells in the outcome stratum.

| **Outcome** | **Predictor** | **OR (95% CI)** | **p** |
| --- | --- | --- | --- |
| Familiarity with PGx (5-level ordered) | Female (vs male) | 0.84 (0.51–1.36) | 0.467 |
| Familiarity with PGx (5-level ordered) | Age (per 1 year) | 0.97 (0.95–0.99) | 0.009 |
| Familiarity with PGx (5-level ordered) | Saudi nationality (vs non-Saudi) | 0.45 (0.16–1.24) | 0.123 |
| Familiarity with PGx (5-level ordered) | High school or less (vs Bachelor) | 1.47 (0.81–2.69) | 0.204 |
| Familiarity with PGx (5-level ordered) | Diploma (vs Bachelor) | 1.45 (0.75–2.80) | 0.273 |
| Familiarity with PGx (5-level ordered) | Master (vs Bachelor) | 1.97 (0.81–4.79) | 0.134 |
| Familiarity with PGx (5-level ordered) | Doctorate (vs Bachelor) | 1.58 (0.59–4.19) | 0.36 |
| Familiarity with PGx (5-level ordered) | Mecca region (vs Riyadh) | 1.16 (0.67–1.98) | 0.598 |
| Familiarity with PGx (5-level ordered) | Other regions (vs Riyadh) | 0.97 (0.62–1.53) | 0.905 |
| Familiarity with PGx (5-level ordered) | Private insurance (vs no) | 1.58 (0.90–2.76) | 0.111 |
| Familiarity with PGx (5-level ordered) | Private facility (vs government) | 0.74 (0.45–1.23) | 0.246 |
| Familiarity with PGx (5-level ordered) | Self-rated good/excellent (vs fair/poor) | 0.81 (0.36–1.80) | 0.604 |
| Familiarity with PGx (5-level ordered) | Chronic disease (vs none) | 0.70 (0.45–1.11) | 0.134 |
| Familiarity with PGx (5-level ordered) | Prior genetic test (vs no) | 3.77 (1.40–10.17) | 0.009 |
| High concern: privacy / unauthorized data sharing (Q14) | Intercept | 5.74 (0.87–37.79) | 0.069 |
| High concern: privacy / unauthorized data sharing (Q14) | Female (vs male) | 0.97 (0.55–1.69) | 0.903 |
| High concern: privacy / unauthorized data sharing (Q14) | Age (per 1 year) | 0.98 (0.95–1.00) | 0.086 |
| High concern: privacy / unauthorized data sharing (Q14) | Saudi nationality (vs non-Saudi) | 0.38 (0.11–1.24) | 0.109 |
| High concern: privacy / unauthorized data sharing (Q14) | High school or less (vs Bachelor) | 1.73 (0.87–3.44) | 0.12 |
| High concern: privacy / unauthorized data sharing (Q14) | Diploma (vs Bachelor) | 1.32 (0.60–2.89) | 0.495 |
| High concern: privacy / unauthorized data sharing (Q14) | Master (vs Bachelor) | 0.95 (0.32–2.82) | 0.922 |
| High concern: privacy / unauthorized data sharing (Q14) | Doctorate (vs Bachelor) | 2.01 (0.66–6.12) | 0.217 |
| High concern: privacy / unauthorized data sharing (Q14) | Mecca region (vs Riyadh) | 1.18 (0.64–2.19) | 0.591 |
| High concern: privacy / unauthorized data sharing (Q14) | Other regions (vs Riyadh) | 1.00 (0.59–1.71) | 0.989 |
| High concern: privacy / unauthorized data sharing (Q14) | Private insurance (vs no) | 1.06 (0.57–1.98) | 0.846 |
| High concern: privacy / unauthorized data sharing (Q14) | Private facility (vs government) | 1.61 (0.92–2.82) | 0.094 |
| High concern: privacy / unauthorized data sharing (Q14) | Self-rated good/excellent (vs fair/poor) | 0.26 (0.11–0.64) | 0.004 |
| High concern: privacy / unauthorized data sharing (Q14) | Chronic disease (vs none) | 1.17 (0.68–2.00) | 0.567 |
| High concern: privacy / unauthorized data sharing (Q14) | Prior genetic test (vs no) | 0.55 (0.14–2.18) | 0.396 |
| High concern: privacy / unauthorized data sharing (Q14) | Any PGx familiarity (vs none) | 1.32 (0.84–2.06) | 0.227 |
| High concern: cost / not cost-effective (Q15) | Intercept | 3.42 (0.51–23.04) | 0.207 |
| High concern: cost / not cost-effective (Q15) | Female (vs male) | 1.00 (0.56–1.79) | 0.995 |
| High concern: cost / not cost-effective (Q15) | Age (per 1 year) | 0.99 (0.96–1.02) | 0.439 |
| High concern: cost / not cost-effective (Q15) | Saudi nationality (vs non-Saudi) | 0.53 (0.16–1.70) | 0.286 |
| High concern: cost / not cost-effective (Q15) | High school or less (vs Bachelor) | 1.38 (0.66–2.87) | 0.387 |
| High concern: cost / not cost-effective (Q15) | Diploma (vs Bachelor) | 1.04 (0.44–2.44) | 0.926 |
| High concern: cost / not cost-effective (Q15) | Master (vs Bachelor) | 1.65 (0.56–4.88) | 0.365 |
| High concern: cost / not cost-effective (Q15) | Doctorate (vs Bachelor) | 1.59 (0.50–5.04) | 0.432 |
| High concern: cost / not cost-effective (Q15) | Mecca region (vs Riyadh) | 0.93 (0.48–1.77) | 0.817 |
| High concern: cost / not cost-effective (Q15) | Other regions (vs Riyadh) | 0.96 (0.55–1.67) | 0.892 |
| High concern: cost / not cost-effective (Q15) | Private insurance (vs no) | 1.28 (0.68–2.41) | 0.447 |
| High concern: cost / not cost-effective (Q15) | Private facility (vs government) | 1.48 (0.83–2.64) | 0.184 |
| High concern: cost / not cost-effective (Q15) | Self-rated good/excellent (vs fair/poor) | 0.25 (0.10–0.62) | 0.003 |
| High concern: cost / not cost-effective (Q15) | Chronic disease (vs none) | 0.78 (0.44–1.38) | 0.386 |
| High concern: cost / not cost-effective (Q15) | Prior genetic test (vs no) | — (sparse) | — |
| High concern: cost / not cost-effective (Q15) | Any PGx familiarity (vs none) | 1.01 (0.63–1.60) | 0.975 |
| High concern: medication side effects (Q10) | Intercept | 0.07 (0.01–0.69) | 0.022 |
| High concern: medication side effects (Q10) | Female (vs male) | 1.10 (0.55–2.20) | 0.787 |
| High concern: medication side effects (Q10) | Age (per 1 year) | 1.03 (1.00–1.06) | 0.025 |
| High concern: medication side effects (Q10) | Saudi nationality (vs non-Saudi) | 1.40 (0.33–5.92) | 0.643 |
| High concern: medication side effects (Q10) | High school or less (vs Bachelor) | 1.26 (0.50–3.19) | 0.626 |
| High concern: medication side effects (Q10) | Diploma (vs Bachelor) | 2.18 (0.91–5.23) | 0.081 |
| High concern: medication side effects (Q10) | Master (vs Bachelor) | 0.37 (0.07–1.84) | 0.224 |
| High concern: medication side effects (Q10) | Doctorate (vs Bachelor) | 2.72 (0.77–9.62) | 0.121 |
| High concern: medication side effects (Q10) | Mecca region (vs Riyadh) | 3.62 (1.72–7.65) | 0.001 |
| High concern: medication side effects (Q10) | Other regions (vs Riyadh) | 1.68 (0.83–3.39) | 0.148 |
| High concern: medication side effects (Q10) | Private insurance (vs no) | 1.37 (0.64–2.92) | 0.422 |
| High concern: medication side effects (Q10) | Private facility (vs government) | 1.16 (0.58–2.34) | 0.673 |
| High concern: medication side effects (Q10) | Self-rated good/excellent (vs fair/poor) | 0.26 (0.10–0.68) | 0.006 |
| High concern: medication side effects (Q10) | Chronic disease (vs none) | 1.20 (0.64–2.26) | 0.57 |
| High concern: medication side effects (Q10) | Prior genetic test (vs no) | 2.04 (0.56–7.37) | 0.279 |
| High concern: medication side effects (Q10) | Any PGx familiarity (vs none) | 1.03 (0.59–1.79) | 0.918 |
| High concern: medication ineffectiveness (Q11) | Intercept | 0.46 (0.05–4.19) | 0.493 |
| High concern: medication ineffectiveness (Q11) | Female (vs male) | 0.87 (0.43–1.75) | 0.695 |
| High concern: medication ineffectiveness (Q11) | Age (per 1 year) | 1.00 (0.97–1.03) | 0.805 |
| High concern: medication ineffectiveness (Q11) | Saudi nationality (vs non-Saudi) | 0.62 (0.16–2.43) | 0.49 |
| High concern: medication ineffectiveness (Q11) | High school or less (vs Bachelor) | 1.41 (0.57–3.49) | 0.454 |
| High concern: medication ineffectiveness (Q11) | Diploma (vs Bachelor) | 2.41 (0.96–6.04) | 0.06 |
| High concern: medication ineffectiveness (Q11) | Master (vs Bachelor) | 0.20 (0.02–1.65) | 0.135 |
| High concern: medication ineffectiveness (Q11) | Doctorate (vs Bachelor) | 2.21 (0.58–8.45) | 0.249 |
| High concern: medication ineffectiveness (Q11) | Mecca region (vs Riyadh) | 3.01 (1.42–6.35) | 0.004 |
| High concern: medication ineffectiveness (Q11) | Other regions (vs Riyadh) | 0.86 (0.41–1.79) | 0.682 |
| High concern: medication ineffectiveness (Q11) | Private insurance (vs no) | 2.14 (0.96–4.78) | 0.064 |
| High concern: medication ineffectiveness (Q11) | Private facility (vs government) | 0.65 (0.30–1.41) | 0.274 |
| High concern: medication ineffectiveness (Q11) | Self-rated good/excellent (vs fair/poor) | 0.30 (0.11–0.81) | 0.018 |
| High concern: medication ineffectiveness (Q11) | Chronic disease (vs none) | 2.08 (1.10–3.92) | 0.024 |
| High concern: medication ineffectiveness (Q11) | Prior genetic test (vs no) | 0.62 (0.12–3.29) | 0.576 |
| High concern: medication ineffectiveness (Q11) | Any PGx familiarity (vs none) | 1.16 (0.65–2.08) | 0.608 |
| High concern: test may not identify suitable drug (Q12) | Intercept | 0.14 (0.01–3.03) | 0.208 |
| High concern: test may not identify suitable drug (Q12) | Female (vs male) | 1.37 (0.50–3.74) | 0.54 |
| High concern: test may not identify suitable drug (Q12) | Age (per 1 year) | 0.98 (0.94–1.03) | 0.416 |
| High concern: test may not identify suitable drug (Q12) | Saudi nationality (vs non-Saudi) | 0.86 (0.13–5.73) | 0.876 |
| High concern: test may not identify suitable drug (Q12) | High school or less (vs Bachelor) | 1.49 (0.47–4.67) | 0.495 |
| High concern: test may not identify suitable drug (Q12) | Diploma (vs Bachelor) | 0.92 (0.19–4.35) | 0.914 |
| High concern: test may not identify suitable drug (Q12) | Master (vs Bachelor) | 0.62 (0.07–5.41) | 0.667 |
| High concern: test may not identify suitable drug (Q12) | Doctorate (vs Bachelor) | 1.26 (0.15–10.54) | 0.828 |
| High concern: test may not identify suitable drug (Q12) | Mecca region (vs Riyadh) | 1.52 (0.54–4.31) | 0.429 |
| High concern: test may not identify suitable drug (Q12) | Other regions (vs Riyadh) | 1.19 (0.47–2.97) | 0.714 |
| High concern: test may not identify suitable drug (Q12) | Private insurance (vs no) | 3.67 (1.35–9.96) | 0.011 |
| High concern: test may not identify suitable drug (Q12) | Private facility (vs government) | 0.57 (0.20–1.62) | 0.295 |
| High concern: test may not identify suitable drug (Q12) | Self-rated good/excellent (vs fair/poor) | 0.62 (0.16–2.49) | 0.504 |
| High concern: test may not identify suitable drug (Q12) | Chronic disease (vs none) | 1.88 (0.79–4.47) | 0.153 |
| High concern: test may not identify suitable drug (Q12) | Prior genetic test (vs no) | — (sparse) | — |
| High concern: test may not identify suitable drug (Q12) | Any PGx familiarity (vs none) | 0.89 (0.41–1.91) | 0.763 |
| High concern: incidental findings (Q13) | Intercept | 0.21 (0.02–2.09) | 0.184 |
| High concern: incidental findings (Q13) | Female (vs male) | 1.55 (0.78–3.07) | 0.207 |
| High concern: incidental findings (Q13) | Age (per 1 year) | 0.99 (0.96–1.02) | 0.506 |
| High concern: incidental findings (Q13) | Saudi nationality (vs non-Saudi) | 1.09 (0.27–4.35) | 0.908 |
| High concern: incidental findings (Q13) | High school or less (vs Bachelor) | 1.17 (0.51–2.71) | 0.706 |
| High concern: incidental findings (Q13) | Diploma (vs Bachelor) | 1.06 (0.41–2.73) | 0.909 |
| High concern: incidental findings (Q13) | Master (vs Bachelor) | 2.58 (0.82–8.11) | 0.105 |
| High concern: incidental findings (Q13) | Doctorate (vs Bachelor) | 1.55 (0.43–5.67) | 0.504 |
| High concern: incidental findings (Q13) | Mecca region (vs Riyadh) | 0.45 (0.21–0.95) | 0.037 |
| High concern: incidental findings (Q13) | Other regions (vs Riyadh) | 0.38 (0.19–0.74) | 0.005 |
| High concern: incidental findings (Q13) | Private insurance (vs no) | 0.97 (0.46–2.07) | 0.941 |
| High concern: incidental findings (Q13) | Private facility (vs government) | 1.24 (0.63–2.45) | 0.528 |
| High concern: incidental findings (Q13) | Self-rated good/excellent (vs fair/poor) | 1.28 (0.39–4.13) | 0.683 |
| High concern: incidental findings (Q13) | Chronic disease (vs none) | 1.80 (0.97–3.31) | 0.061 |
| High concern: incidental findings (Q13) | Prior genetic test (vs no) | — (sparse) | — |
| High concern: incidental findings (Q13) | Any PGx familiarity (vs none) | 0.85 (0.50–1.44) | 0.535 |
| High concern: accuracy / reliability (Q16) | Intercept | 0.32 (0.03–3.09) | 0.324 |
| High concern: accuracy / reliability (Q16) | Female (vs male) | 1.32 (0.62–2.78) | 0.47 |
| High concern: accuracy / reliability (Q16) | Age (per 1 year) | 0.99 (0.96–1.02) | 0.483 |
| High concern: accuracy / reliability (Q16) | Saudi nationality (vs non-Saudi) | 0.67 (0.18–2.50) | 0.556 |
| High concern: accuracy / reliability (Q16) | High school or less (vs Bachelor) | 1.38 (0.53–3.57) | 0.511 |
| High concern: accuracy / reliability (Q16) | Diploma (vs Bachelor) | 1.63 (0.60–4.45) | 0.341 |
| High concern: accuracy / reliability (Q16) | Master (vs Bachelor) | 3.02 (0.95–9.59) | 0.061 |
| High concern: accuracy / reliability (Q16) | Doctorate (vs Bachelor) | 2.80 (0.71–10.99) | 0.14 |
| High concern: accuracy / reliability (Q16) | Mecca region (vs Riyadh) | 0.72 (0.31–1.65) | 0.432 |
| High concern: accuracy / reliability (Q16) | Other regions (vs Riyadh) | 0.78 (0.38–1.60) | 0.492 |
| High concern: accuracy / reliability (Q16) | Private insurance (vs no) | 1.00 (0.46–2.17) | 0.991 |
| High concern: accuracy / reliability (Q16) | Private facility (vs government) | 1.97 (0.95–4.05) | 0.067 |
| High concern: accuracy / reliability (Q16) | Self-rated good/excellent (vs fair/poor) | 0.55 (0.19–1.58) | 0.268 |
| High concern: accuracy / reliability (Q16) | Chronic disease (vs none) | 1.67 (0.85–3.27) | 0.135 |
| High concern: accuracy / reliability (Q16) | Prior genetic test (vs no) | — (sparse) | — |
| High concern: accuracy / reliability (Q16) | Any PGx familiarity (vs none) | 1.11 (0.62–2.00) | 0.727 |
| Perceived employment disadvantage (Q17) | Intercept | 0.22 (0.03–1.66) | 0.14 |
| Perceived employment disadvantage (Q17) | Female (vs male) | 1.46 (0.84–2.52) | 0.182 |
| Perceived employment disadvantage (Q17) | Age (per 1 year) | 1.01 (0.99–1.03) | 0.48 |
| Perceived employment disadvantage (Q17) | Saudi nationality (vs non-Saudi) | 3.13 (0.72–13.69) | 0.129 |
| Perceived employment disadvantage (Q17) | High school or less (vs Bachelor) | 0.88 (0.43–1.77) | 0.717 |
| Perceived employment disadvantage (Q17) | Diploma (vs Bachelor) | 0.78 (0.36–1.68) | 0.527 |
| Perceived employment disadvantage (Q17) | Master (vs Bachelor) | 0.74 (0.24–2.27) | 0.6 |
| Perceived employment disadvantage (Q17) | Doctorate (vs Bachelor) | 1.33 (0.44–4.06) | 0.616 |
| Perceived employment disadvantage (Q17) | Mecca region (vs Riyadh) | 0.67 (0.37–1.23) | 0.195 |
| Perceived employment disadvantage (Q17) | Other regions (vs Riyadh) | 0.68 (0.41–1.14) | 0.142 |
| Perceived employment disadvantage (Q17) | Private insurance (vs no) | 0.72 (0.38–1.37) | 0.315 |
| Perceived employment disadvantage (Q17) | Private facility (vs government) | 1.20 (0.69–2.08) | 0.512 |
| Perceived employment disadvantage (Q17) | Self-rated good/excellent (vs fair/poor) | 0.80 (0.33–1.94) | 0.626 |
| Perceived employment disadvantage (Q17) | Chronic disease (vs none) | 0.98 (0.58–1.65) | 0.93 |
| Perceived employment disadvantage (Q17) | Prior genetic test (vs no) | 0.55 (0.14–2.12) | 0.384 |
| Perceived employment disadvantage (Q17) | Any PGx familiarity (vs none) | 0.70 (0.45–1.07) | 0.099 |
| Perceived health-insurance disadvantage (Q18) | Intercept | 0.34 (0.05–2.19) | 0.255 |
| Perceived health-insurance disadvantage (Q18) | Female (vs male) | 0.74 (0.43–1.28) | 0.282 |
| Perceived health-insurance disadvantage (Q18) | Age (per 1 year) | 1.04 (1.01–1.06) | 0.004 |
| Perceived health-insurance disadvantage (Q18) | Saudi nationality (vs non-Saudi) | 1.37 (0.41–4.63) | 0.611 |
| Perceived health-insurance disadvantage (Q18) | High school or less (vs Bachelor) | 1.87 (0.94–3.74) | 0.076 |
| Perceived health-insurance disadvantage (Q18) | Diploma (vs Bachelor) | 1.30 (0.60–2.80) | 0.501 |
| Perceived health-insurance disadvantage (Q18) | Master (vs Bachelor) | 1.20 (0.42–3.42) | 0.734 |
| Perceived health-insurance disadvantage (Q18) | Doctorate (vs Bachelor) | 0.86 (0.29–2.58) | 0.793 |
| Perceived health-insurance disadvantage (Q18) | Mecca region (vs Riyadh) | 1.41 (0.77–2.61) | 0.268 |
| Perceived health-insurance disadvantage (Q18) | Other regions (vs Riyadh) | 0.77 (0.45–1.32) | 0.338 |
| Perceived health-insurance disadvantage (Q18) | Private insurance (vs no) | 1.29 (0.67–2.47) | 0.452 |
| Perceived health-insurance disadvantage (Q18) | Private facility (vs government) | 0.78 (0.43–1.42) | 0.424 |
| Perceived health-insurance disadvantage (Q18) | Self-rated good/excellent (vs fair/poor) | 0.40 (0.17–0.98) | 0.045 |
| Perceived health-insurance disadvantage (Q18) | Chronic disease (vs none) | 0.84 (0.50–1.43) | 0.532 |
| Perceived health-insurance disadvantage (Q18) | Prior genetic test (vs no) | 0.88 (0.26–2.97) | 0.843 |
| Perceived health-insurance disadvantage (Q18) | Any PGx familiarity (vs none) | 0.95 (0.60–1.48) | 0.807 |
| Cost a concern if self-paid (Q19) | Intercept | 0.98 (0.12–8.23) | 0.984 |
| Cost a concern if self-paid (Q19) | Female (vs male) | 0.94 (0.52–1.71) | 0.847 |
| Cost a concern if self-paid (Q19) | Age (per 1 year) | 1.01 (0.99–1.04) | 0.34 |
| Cost a concern if self-paid (Q19) | Saudi nationality (vs non-Saudi) | 2.06 (0.49–8.65) | 0.325 |
| Cost a concern if self-paid (Q19) | High school or less (vs Bachelor) | 2.26 (1.02–5.02) | 0.045 |
| Cost a concern if self-paid (Q19) | Diploma (vs Bachelor) | 1.09 (0.50–2.41) | 0.825 |
| Cost a concern if self-paid (Q19) | Master (vs Bachelor) | 8.20 (1.01–66.32) | 0.049 |
| Cost a concern if self-paid (Q19) | Doctorate (vs Bachelor) | 2.98 (0.71–12.41) | 0.134 |
| Cost a concern if self-paid (Q19) | Mecca region (vs Riyadh) | 1.03 (0.53–2.00) | 0.92 |
| Cost a concern if self-paid (Q19) | Other regions (vs Riyadh) | 0.87 (0.51–1.50) | 0.624 |
| Cost a concern if self-paid (Q19) | Private insurance (vs no) | 0.81 (0.41–1.60) | 0.541 |
| Cost a concern if self-paid (Q19) | Private facility (vs government) | 1.58 (0.86–2.90) | 0.138 |
| Cost a concern if self-paid (Q19) | Self-rated good/excellent (vs fair/poor) | 0.64 (0.22–1.85) | 0.409 |
| Cost a concern if self-paid (Q19) | Chronic disease (vs none) | 1.24 (0.69–2.21) | 0.47 |
| Cost a concern if self-paid (Q19) | Prior genetic test (vs no) | 0.48 (0.14–1.64) | 0.241 |
| Cost a concern if self-paid (Q19) | Any PGx familiarity (vs none) | 0.98 (0.62–1.56) | 0.933 |
| More willing if free/covered by insurance (Q20) | Intercept | 0.26 (0.02–4.27) | 0.344 |
| More willing if free/covered by insurance (Q20) | Female (vs male) | 1.05 (0.51–2.17) | 0.887 |
| More willing if free/covered by insurance (Q20) | Age (per 1 year) | 1.02 (0.99–1.06) | 0.193 |
| More willing if free/covered by insurance (Q20) | Saudi nationality (vs non-Saudi) | 8.19 (1.03–65.00) | 0.047 |
| More willing if free/covered by insurance (Q20) | High school or less (vs Bachelor) | 1.15 (0.48–2.75) | 0.749 |
| More willing if free/covered by insurance (Q20) | Diploma (vs Bachelor) | 1.21 (0.45–3.29) | 0.702 |
| More willing if free/covered by insurance (Q20) | Master (vs Bachelor) | — (sparse) | — |
| More willing if free/covered by insurance (Q20) | Doctorate (vs Bachelor) | 12.89 (0.89–185.77) | 0.06 |
| More willing if free/covered by insurance (Q20) | Mecca region (vs Riyadh) | 4.24 (1.44–12.50) | 0.009 |
| More willing if free/covered by insurance (Q20) | Other regions (vs Riyadh) | 1.00 (0.54–1.86) | 0.998 |
| More willing if free/covered by insurance (Q20) | Private insurance (vs no) | 1.04 (0.45–2.39) | 0.934 |
| More willing if free/covered by insurance (Q20) | Private facility (vs government) | 0.90 (0.44–1.86) | 0.781 |
| More willing if free/covered by insurance (Q20) | Self-rated good/excellent (vs fair/poor) | 0.71 (0.19–2.67) | 0.617 |
| More willing if free/covered by insurance (Q20) | Chronic disease (vs none) | 2.24 (0.99–5.09) | 0.054 |
| More willing if free/covered by insurance (Q20) | Prior genetic test (vs no) | 0.88 (0.17–4.61) | 0.885 |
| More willing if free/covered by insurance (Q20) | Any PGx familiarity (vs none) | 0.92 (0.52–1.63) | 0.769 |
| Prior notification by provider (Q21, agree) | Intercept | — (sparse) | — |
| Prior notification by provider (Q21, agree) | Female (vs male) | 0.34 (0.09–1.35) | 0.125 |
| Prior notification by provider (Q21, agree) | Age (per 1 year) | 1.00 (0.95–1.05) | 0.913 |
| Prior notification by provider (Q21, agree) | Saudi nationality (vs non-Saudi) | 6.22 (0.39–99.77) | 0.197 |
| Prior notification by provider (Q21, agree) | High school or less (vs Bachelor) | 0.74 (0.22–2.49) | 0.631 |
| Prior notification by provider (Q21, agree) | Diploma (vs Bachelor) | 1.41 (0.29–6.94) | 0.673 |
| Prior notification by provider (Q21, agree) | Master (vs Bachelor) | — (sparse) | — |
| Prior notification by provider (Q21, agree) | Doctorate (vs Bachelor) | — (sparse) | — |
| Prior notification by provider (Q21, agree) | Mecca region (vs Riyadh) | 1.39 (0.34–5.77) | 0.65 |
| Prior notification by provider (Q21, agree) | Other regions (vs Riyadh) | 0.43 (0.17–1.10) | 0.079 |
| Prior notification by provider (Q21, agree) | Private insurance (vs no) | 0.69 (0.18–2.57) | 0.578 |
| Prior notification by provider (Q21, agree) | Private facility (vs government) | 3.16 (0.87–11.43) | 0.08 |
| Prior notification by provider (Q21, agree) | Self-rated good/excellent (vs fair/poor) | — (sparse) | — |
| Prior notification by provider (Q21, agree) | Chronic disease (vs none) | 1.25 (0.39–3.95) | 0.707 |
| Prior notification by provider (Q21, agree) | Prior genetic test (vs no) | 0.60 (0.06–5.84) | 0.662 |
| Prior notification by provider (Q21, agree) | Any PGx familiarity (vs none) | 1.84 (0.80–4.26) | 0.153 |
| Specific consent when bundled with blood work (Q22, agree) | Intercept | 1.84 (0.15–23.05) | 0.636 |
| Specific consent when bundled with blood work (Q22, agree) | Female (vs male) | 0.82 (0.40–1.70) | 0.599 |
| Specific consent when bundled with blood work (Q22, agree) | Age (per 1 year) | 1.03 (0.99–1.06) | 0.101 |
| Specific consent when bundled with blood work (Q22, agree) | Saudi nationality (vs non-Saudi) | 1.81 (0.37–8.90) | 0.466 |
| Specific consent when bundled with blood work (Q22, agree) | High school or less (vs Bachelor) | 0.53 (0.24–1.21) | 0.132 |
| Specific consent when bundled with blood work (Q22, agree) | Diploma (vs Bachelor) | 0.61 (0.23–1.57) | 0.303 |
| Specific consent when bundled with blood work (Q22, agree) | Master (vs Bachelor) | 0.87 (0.18–4.32) | 0.865 |
| Specific consent when bundled with blood work (Q22, agree) | Doctorate (vs Bachelor) | 0.91 (0.17–4.75) | 0.908 |
| Specific consent when bundled with blood work (Q22, agree) | Mecca region (vs Riyadh) | 2.51 (0.94–6.71) | 0.065 |
| Specific consent when bundled with blood work (Q22, agree) | Other regions (vs Riyadh) | 0.90 (0.47–1.71) | 0.742 |
| Specific consent when bundled with blood work (Q22, agree) | Private insurance (vs no) | 1.52 (0.62–3.74) | 0.36 |
| Specific consent when bundled with blood work (Q22, agree) | Private facility (vs government) | 0.90 (0.43–1.92) | 0.793 |
| Specific consent when bundled with blood work (Q22, agree) | Self-rated good/excellent (vs fair/poor) | 0.90 (0.24–3.30) | 0.872 |
| Specific consent when bundled with blood work (Q22, agree) | Chronic disease (vs none) | 0.67 (0.33–1.33) | 0.253 |
| Specific consent when bundled with blood work (Q22, agree) | Prior genetic test (vs no) | 2.32 (0.28–19.30) | 0.437 |
| Specific consent when bundled with blood work (Q22, agree) | Any PGx familiarity (vs none) | 1.02 (0.57–1.81) | 0.954 |
| Attitude item Q1: more positive response (ordered logistic) | Female (vs male) | 0.60 (0.37–0.98) | 0.04 |
| Attitude item Q1: more positive response (ordered logistic) | Age (per 1 year) | 0.99 (0.97–1.01) | 0.381 |
| Attitude item Q1: more positive response (ordered logistic) | Saudi nationality (vs non-Saudi) | 2.26 (0.81–6.32) | 0.12 |
| Attitude item Q1: more positive response (ordered logistic) | High school or less (vs Bachelor) | 1.15 (0.63–2.10) | 0.652 |
| Attitude item Q1: more positive response (ordered logistic) | Diploma (vs Bachelor) | 1.17 (0.59–2.32) | 0.648 |
| Attitude item Q1: more positive response (ordered logistic) | Master (vs Bachelor) | 1.92 (0.80–4.60) | 0.142 |
| Attitude item Q1: more positive response (ordered logistic) | Doctorate (vs Bachelor) | 3.24 (1.18–8.93) | 0.023 |
| Attitude item Q1: more positive response (ordered logistic) | Mecca region (vs Riyadh) | 1.10 (0.65–1.86) | 0.718 |
| Attitude item Q1: more positive response (ordered logistic) | Other regions (vs Riyadh) | 0.65 (0.42–1.01) | 0.057 |
| Attitude item Q1: more positive response (ordered logistic) | Private insurance (vs no) | 0.91 (0.52–1.57) | 0.726 |
| Attitude item Q1: more positive response (ordered logistic) | Private facility (vs government) | 1.14 (0.71–1.84) | 0.583 |
| Attitude item Q1: more positive response (ordered logistic) | Self-rated good/excellent (vs fair/poor) | 0.85 (0.38–1.90) | 0.693 |
| Attitude item Q1: more positive response (ordered logistic) | Chronic disease (vs none) | 1.62 (1.02–2.56) | 0.041 |
| Attitude item Q1: more positive response (ordered logistic) | Prior genetic test (vs no) | 1.81 (0.62–5.27) | 0.279 |
| Attitude item Q1: more positive response (ordered logistic) | Any PGx familiarity (vs none) | 1.15 (0.79–1.68) | 0.478 |
| Attitude item Q1: more positive response (ordered logistic) | Female (vs male) | 0.70 (0.43–1.12) | 0.139 |
| Attitude item Q1: more positive response (ordered logistic) | Age (per 1 year) | 0.99 (0.97–1.01) | 0.534 |
| Attitude item Q1: more positive response (ordered logistic) | Saudi nationality (vs non-Saudi) | 1.63 (0.58–4.57) | 0.351 |
| Attitude item Q1: more positive response (ordered logistic) | High school or less (vs Bachelor) | 1.23 (0.68–2.23) | 0.493 |
| Attitude item Q1: more positive response (ordered logistic) | Diploma (vs Bachelor) | 1.09 (0.55–2.16) | 0.804 |
| Attitude item Q1: more positive response (ordered logistic) | Master (vs Bachelor) | 1.88 (0.78–4.51) | 0.158 |
| Attitude item Q1: more positive response (ordered logistic) | Doctorate (vs Bachelor) | 2.92 (1.09–7.79) | 0.033 |
| Attitude item Q1: more positive response (ordered logistic) | Mecca region (vs Riyadh) | 1.08 (0.65–1.82) | 0.76 |
| Attitude item Q1: more positive response (ordered logistic) | Other regions (vs Riyadh) | 0.78 (0.50–1.21) | 0.264 |
| Attitude item Q1: more positive response (ordered logistic) | Private insurance (vs no) | 0.70 (0.41–1.21) | 0.204 |
| Attitude item Q1: more positive response (ordered logistic) | Private facility (vs government) | 1.50 (0.93–2.42) | 0.097 |
| Attitude item Q6: more positive response (ordered logistic) | Self-rated good/excellent (vs fair/poor) | 0.78 (0.36–1.71) | 0.534 |
| Attitude item Q6: more positive response (ordered logistic) | Chronic disease (vs none) | 1.63 (1.03–2.56) | 0.035 |
| Attitude item Q6: more positive response (ordered logistic) | Prior genetic test (vs no) | 1.67 (0.56–5.00) | 0.362 |
| Attitude item Q6: more positive response (ordered logistic) | Any PGx familiarity (vs none) | 1.11 (0.76–1.62) | 0.577 |
| Overall favorable attitude (mean Likert ≥ 4) | Intercept | 0.56 (0.09–3.59) | 0.537 |
| Overall favorable attitude (mean Likert ≥ 4) | Female (vs male) | 0.93 (0.54–1.58) | 0.776 |
| Overall favorable attitude (mean Likert ≥ 4) | Age (per 1 year) | 1.00 (0.98–1.03) | 0.905 |
| Overall favorable attitude (mean Likert ≥ 4) | Saudi nationality (vs non-Saudi) | 2.85 (0.84–9.66) | 0.092 |
| Overall favorable attitude (mean Likert ≥ 4) | High school or less (vs Bachelor) | 1.91 (0.96–3.80) | 0.067 |
| Overall favorable attitude (mean Likert ≥ 4) | Diploma (vs Bachelor) | 1.37 (0.65–2.88) | 0.405 |
| Overall favorable attitude (mean Likert ≥ 4) | Master (vs Bachelor) | 2.30 (0.73–7.27) | 0.157 |
| Overall favorable attitude (mean Likert ≥ 4) | Doctorate (vs Bachelor) | 3.92 (1.16–13.28) | 0.028 |
| Overall favorable attitude (mean Likert ≥ 4) | Mecca region (vs Riyadh) | 1.77 (0.96–3.27) | 0.066 |
| Overall favorable attitude (mean Likert ≥ 4) | Other regions (vs Riyadh) | 0.93 (0.57–1.54) | 0.789 |
| Overall favorable attitude (mean Likert ≥ 4) | Private insurance (vs no) | 0.77 (0.42–1.42) | 0.405 |
| Overall favorable attitude (mean Likert ≥ 4) | Private facility (vs government) | 1.15 (0.67–1.99) | 0.613 |
| Overall favorable attitude (mean Likert ≥ 4) | Self-rated good/excellent (vs fair/poor) | 0.62 (0.25–1.54) | 0.301 |
| Overall favorable attitude (mean Likert ≥ 4) | Chronic disease (vs none) | 1.31 (0.78–2.20) | 0.305 |
| Overall favorable attitude (mean Likert ≥ 4) | Prior genetic test (vs no) | 2.15 (0.55–8.42) | 0.274 |
| Overall favorable attitude (mean Likert ≥ 4) | Any PGx familiarity (vs none) | 0.93 (0.61–1.42) | 0.735 |

*OR, odds ratio; CI, confidence interval. P-values are two-tailed. "— (sparse)" indicates a coefficient that cannot be reliably estimated owing to near-complete separation (zero or near-zero events in the outcome stratum) or non-convergence, typically because the predictor has very few participants in the relevant cell (e.g., n = 13 with prior genetic testing; n = 17 non-Saudi). AUCs (binary outcomes only): privacy 0.66, cost 0.65, willingness 0.69, favorable attitude 0.64; ordered logistic models do not produce AUCs.*
