# Supplementary material for: Public awareness, attitudes, and concerns regarding pharmacogenomics in the Kingdom of Saudi Arabia: an exploratory online cross-sectional survey
Source: Front Public Health. 2026 Aug 24;14:1887596. doi: 10.3389/fpubh.2026.1887596 (PMC13547504; doi:10.3389/fpubh.2026.1887596)
Supplement: Supplementary file 2 [file Table_2.docx]

**Awareness, Attitudes, and Concerns Regarding Pharmacogenomics in the Kingdom of Saudi Arabia: An Exploratory Online Cross-Sectional Survey**

**Appendix B. Questionnaire on Pharmacogenetic Testing.**
(English version; administered to the general public in Saudi Arabia in Arabic)

### **Section 1: Eligibility Determination and Consent to Participate in the Study and Publish Its Results**

### All data collected will be treated with strict confidentiality and used solely for scientific research purposes, in accordance with ethical standards. You may skip any question you feel uncomfortable answering.

1. **Are you 18 years of age or older?**
   - ☐ Yes
   - ☐ No
2. **Do you agree to participate in this study and to the publication of its results?**
   - ☐ Yes
   - ☐ No

### **Section 2. Sociodemographic and Healthcare Characteristics**

1. **Age in years**
   - ………..
2. **Gender**
   - ☐ Male
   - ☐ Female
3. **Nationality**
   - ☐ Saudi
   - ☐ Non-Saudi
4. **Educational qualification**
   - ☐ High school or less
   - ☐ Diploma
   - ☐ Bachelor’s degree
   - ☐ Master’s degree or equivalent
   - ☐ Doctorate or equivalent
5. **Province of residence**
   - ☐ Riyadh Province
   - ☐ Mecca Province
   - ☐ Medina Province
   - ☐ Eastern Province
   - ☐ Al-Qassim Province
   - ☐ Asir Province
   - ☐ Tabuk Province
   - ☐ Ḥa’il Province
   - ☐ Northern Borders Province
   - ☐ Jazan Province
   - ☐ Najran Province
   - ☐ Al-Bahah Province
   - ☐ Al-Jawf Province
6. **Do you have private health insurance?**
   - ☐ Yes
   - ☐ No
7. **Type of healthcare facility you usually visit when you need medical care**
   - ☐ Government hospital/clinic
   - ☐ Private hospital/clinic
8. **How would you rate your current health?**
   - ☐ Excellent (You feel very healthy and have no major health problems)
   - Good (Your health is generally good with some minor issues)
   - Fair (You have some health problems that affect your daily life)
   - Poor (You have major health problems that significantly affect your life)
9. **Do you have any chronic diseases?**
   - ☐ Yes
   - ☐ No
10. **If your answer to the previous question was Yes, what chronic diseases/conditions do you suffer from?**
    - ☐ Diabetes mellitus
    - ☐ Cardiovascular / hypertension
    - ☐ Respiratory diseases (e.g., asthma, COPD)
    - ☐ Rheumatologic diseases (e.g., arthritis)
    - ☐ Gastrointestinal diseases
    - ☐ Neurologic diseases/migraine
    - ☐ Psychiatric disorders
    - ☐ Dermatologic diseases
    - ☐ Hematologic diseases
    - ☐ Bacterial infections (antibiotics)
    - ☐ Cancer therapy
    - ☐ Gynecologic/hormonal disorders
    - ☐ Other: _____________

### **Section 3. Self-Reported History of Genetic Testing and Familiarity with PGx**

1. **Have you ever undergone any genetic test (for any reason)?**
   - ☐ Yes
   - ☐ No
2. **How would you rate your Familiarity with the term “pharmacogenomics” *(The science that studies how your genes affect your response to medications)***
   - ☐ Completely unfamiliar
   - ☐ Somewhat unfamiliar
   - ☐ Moderately familiar
   - ☐ Very familiar
   - ☐ Extremely familiar
3. **Has your healthcare provider ever discussed pharmacogenomics concepts with you?**
   - ☐ Yes
   - ☐ No

### **Section 4. Attitudes toward pharmacogenetic testing**

For Items 1–9 below, please indicate your level of agreement:

**Response options (5-point Likert scale):**

- ☐ Strongly disagree
- ☐ Disagree
- ☐ Neither agree nor disagree
- ☐ Agree
- ☐ Strongly agree

1. I feel comfortable about the pharmacogenetic tests that might be offered to me, if they could predict if a medication would work for my condition.
2. I feel comfortable about the pharmacogenetic tests that might be offered to me, if they could predict the correct dose of the medication that I needed.
3. I feel comfortable about the pharmacogenetic tests that might be offered to me, if they could predict side effects.
4. I feel comfortable about the pharmacogenetic tests that might be offered to me, if they could explain a family history of medication side effects or no response.
5. I believe that using genetic testing is essential for patients taking five or more medications to identify suitable drugs, reduce drug interactions, side effects, or medication ineffectiveness.
6. **If my healthcare provider recommended it, I would feel comfortable getting a pharmacogenetic test.**
7. Do you think pharmacogenetic testing will increase your adherence to taking medications?
8. I believe that pharmacogenetic testing will contribute to improved healthcare outcomes and more efficient healthcare spending.
9. Do you think that knowing more about your genetic predisposition to a certain disease would make you change your lifestyle in a way that reduces the risk of developing that disease?

### **Section 5. Concerns, Uncertainties, and Preferences Regarding Pharmacogenetic Testing**

**Part A – Level of concern (3-point scale)**

For each of the following, please indicate your level of concern:

Response options:

- ☐ Low concern
- ☐ No concern
- ☐ High concern

10. How concerned are you about experiencing side effects from medications?
11. How concerned are you about medications being ineffective for you?
12. How concerned are you that a pharmacogenetic test might not identify a suitable medication for you (for example, that no available drug would work or that severe side effects would be likely)?
13. How concerned are you that a pharmacogenetic test might, by chance, reveal additional risk factors for another disease that you were unaware of?
14. How concerned are you that the results of a pharmacogenetic test might be passed on to unauthorized persons?
15. How concerned are you that pharmacogenetic testing might be expensive and not cost-effective?
16. How concerned are you about the accuracy and reliability of pharmacogenetic testing?

**Part B – Yes/No concerns**

Please answer Yes or No:

17. In case an unfavorable test result should be disclosed, do you believe that you would be disadvantaged at work or when seeking a job?
18. In case an unfavorable test result should be disclosed, do you believe that you would be disadvantaged by health insurance companies?
19. If you had to pay for pharmacogenetic testing yourself, would financial cost be one of your concerns about undergoing the test?
20. If pharmacogenetic testing were free or covered by insurance, would you be more willing to undergo it to improve medication selection and reduce treatment-related risks?

**Part C – Communication and consent preferences**

Response options:

- ☐ Agree
- ☐ Disagree

21. It is important that my healthcare provider informs me about pharmacogenetic tests before any are performed.
22. If pharmacogenetic tests were included as part of my usual blood work, it is important that my healthcare provider obtains my separate, specific approval for those pharmacogenetic tests.
