# Supplementary material for: Public awareness, attitudes, and concerns regarding pharmacogenomics in the Kingdom of Saudi Arabia: an exploratory online cross-sectional survey
Source: Front Public Health. 2026 Aug 24;14:1887596. doi: 10.3389/fpubh.2026.1887596 (PMC13547504; doi:10.3389/fpubh.2026.1887596)
Supplement: Supplementary file 3 [file Table_3.docx]

**الوعي والمواقف والمخاوف حول علم الصيدلة الوراثي في المملكة العربية السعودية: استطلاع مقطعي استكشافي عبر الإنترنت**

**القسم الأول: تحديد الأهلية و الموافقة على المشاركة في الدراسة ونشر نتائجها.**

**جميع البيانات التي يتم جمعها ستُعامل بسرية تامة وتُستخدم لأغراض البحث العلمي فقط، وفقًا للمعايير الأخلاقية. يمكنك تخطي أي سؤال تشعر بعدم الراحة في الإجابة عليه.**

1. **هل عمرك 18 سنة أو أكثر؟**
   - نعم
   - لا
2. **هل توافق على المشاركة في هذه الدراسة ونشر نتائجها؟**
   - نعم
   - لا

**القسم الثاني: الأسئلة العامة التي تهدف إلى معرفة البيانات الاجتماعية والديموغرافية والصحية.**

1. **ماهو عمرك؟ (الرجاء كتابته رقمًا مثال: 30)**
   - …………
2. **ما هو جنسك؟**
   - ذكر
   - أنثى
3. **ماهي جنسيتك؟**
   - سعودي
   - غير سعودي
4. **ماهو مؤهلك العلمي؟**
   - شهادة الثانوية العامة أو أقل
   - شهادة الدبلوم
   - شهادة البكالوريوس
   - شهادة الماجستير وما يعادلها
   - شهادة الدكتوراه وما يعادلها
5. **ما هي منطقتك الإدارية ؟**
   - منطقة مكة المكرمة
   - منطقة المدينة المنورة
   - منطقة الرياض
   - منطقة الشرقية
   - منطقة عسير
   - منطقة تبوك
   - منطقة الجوف
   - منطقة جازان
   - منطقة حائل
   - منطقة القصيم
   - منطقة الباحة
   - منطقة نجران
   - منطقة الحدود الشمالية
6. **هل لديك تأمين صحي خاص؟**
   - نعم
   - لا
7. **ماهو نوع المنشأة الصحية الذي تتلقى منها رعايتك الصحية عادةً؟**
   - حكومي
   - خاص
8. **ماهو تقييمك لصحتك العامة؟**
   - ممتاز (تشعر بصحة جيدة جدًا ولا تعاني من مشاكل صحية كبيرة)
   - جيد (صحتك جيدة بشكل عام مع بعض المشاكل البسيطة)
   - مقبول (تعاني من بعض المشاكل الصحية التي تؤثر على حياتك اليومية)
   - ضعيف (تعاني من مشاكل صحية كبيرة تؤثر بشكل كبير على حياتك)
9. **هل لديك امراض مزمنة؟**
   - نعم
   - لا
10. **إذا كانت اجابتك للسؤال السابق بنعم، ماهي الأمراض المزمنة التي لديك؟**
    - داء السكري (نوع أول، نوع ثاني، سكر الحمل)
    - أمراض القلب والأوعية الدموية (فشل القلب، الجلطات الدموية مثل تجلّط الأوردة العميقة او الانسداد الرئوي)
    - ارتفاع ضغط الدم
    - السكتات الدماغية
    - أمراض الكلى
    - أمراض الكبد
    - أمراض الجهاز التنفسي (الربو،مرض الانسداد الرئوي المزمن)
    - أمراض الجهاز الهضمي
    - التهاب المفاصل الروماتويدي
    - اضطرابات وأمراض الجهاز العصبي
    - الأورام السرطانية
    - الأمراض والاضطرابات النفسية (الإكتئاب، ثنائي القطب)
    - أخرى: ...............

**القسم الثالث: التاريخ الذاتي لاختبارات الجينات والإلمام بالدواء الجيني (PGx)**

1. **هل خضعت من قبل لأي اختبار جيني (لأي سبب)؟**

- نعم
- لا

2. **هل سمعت من قبل بمصطلح "علم الصيدلة الجيني"؟ (علم يدرس طريقة تأثير الجينات على استجابتك للأدوية)**

- غير مألوف تمامًا
- غير مألوف إلى حد ما
- مألوف بشكل متوسط
- مألوف جدًا
- مألوف بشكل كبير جدًا

3. **هل ناقش معك مقدم الرعاية الصحية الخاص بك مفاهيم الدواء الجيني (Pharmacogenomics) من قبل؟**

- نعم
- لا

**القسم الرابع: الاتجاهات والمواقف نحو علم الصيدلة الجيني**

**يهدف هذا القسم إلى تقييم الاتجاهات والمواقف العامة نحو علم الوراثة الدوائي**

1. **سوف أشعر بالرضا تجاه إجراء اختبارات جينية دوائية إذا كانت تستطيع التنبؤ بالدواء المناسب لي ومدى فعاليته.**
   - لا أوافق بشدة
   - لا أوافق
   - محايد
   - أوافق
   - أوافق بشدة
2. **سوف أشعر بالرضا تجاه إجراء الاختبارات الجينية إذا كانت قادرة على اقتراح الجرعة المناسبة من الدواء الملائم لي.**
   - لا أوافق بشدة
   - لا أوافق
   - محايد
   - أوافق
   - أوافق بشدة
3. **سوف أشعر بالرضا بشأن تطبيق الإختبارات الجينية التي تطلب مني إذ كانت قادرة على التنبؤ بالأعراض الجانبية اللي يسببها الدواء.**
   - لا أوافق بشدة
   - لا أوافق
   - محايد
   - أوافق
   - أوافق بشدة
4. **أشعر بالراحة تجاه اختبارات الجينات الدوائية التي يمكن أن تُعرض عليّ، إذا كانت قادرة على تفسير تاريخ عائلي من الآثار الجانبية للأدوية أو عدم الاستجابة لها.**
   - لا أوافق بشدة
   - لا أوافق
   - محايد
   - أوافق
   - أوافق بشدة
5. **أعتقد أن إستخدام الاختبارات الجينية ضروري للمرضى الذين يتناولون 5 أدوية او اكثر للكشف عن الأدوية المناسبة والحد من التعارضات بين الأدوية أو الأعراض الجانبية أو عدم فعالية الأدوية.**
   - لا أوافق بشدة
   - لا أوافق
   - محايد
   - أوافق
   - أوافق بشدة
6. **سأشعر بالراحة تجاه إجراء اختبار الصيدلة الجينية إذا أوصى به مقدم الرعاية الصحية الخاص بي.**
   - لا أوافق بشدة
   - لا أوافق
   - محايد
   - أوافق
   - أوافق بشدة
7. **هل تعتقد أن الاختبارات الجينية المتعلقة بالأدوية قد تساهم في تحسين الالتزام بتناول ادويتك.**
   - لا أوافق بشدة
   - لا أوافق
   - محايد
   - أوافق
   - أوافق بشدة
8. **أعتقد أن تنفيذ إختبارات الصيدلة الجينية في الممارسة السريرية سوف تساهم في تحسين الخدمات الصحية ومخرجات الرعاية وتقليل التكاليف**
   - لا أوافق بشدة
   - لا أوافق
   - محايد
   - أوافق
   - أوافق بشدة
9. **هل تعتقد أن معرفة احتمالية إصابتك بمرض معين بسبب جيناتك ستدفعك لتغيير نمط حياتك لتقليل هذا الخطر؟**
   - لا أوافق بشدة
   - لا أوافق
   - محايد
   - أوافق
   - أوافق بشدة

**القسم الخامس: المخاوف والقلق نحو علم الصيدلة الجيني**

**يهدف هذا القسم إلى قياس القلق والمخاوف والشكوك حول تنفيذ أو تطبيق الاختبارات الجينية.**

**الجزء أ – مستوى القلق (مقياس ثلاثي النقاط(**

بالنسبة لكل مما يلي، يرجى تحديد مستوى قلقك:

خيارات الإجابة:

- قلق منخفض
- لا يوجد قلق
- قلق مرتفع

10. **هل لديك قلق من حدوث الأعراض الجانبية للأدوية؟**
    a. قلق منخفض
    b. قلق شديد
    c. لا يوجد قلق
11. **هل لديك قلق من عدم فعالية الأدوية؟**
    a. قلق منخفض
    b. قلق شديد
    c. لا يوجد قلق
12. **هل تخشى أن اختبار الصيدلة الجينية قد لا يحدد دواءً مناسباً لك، مثل أن الأدوية المتوفرة قد لا تكون فعالة أو قد تسبب آثارًا جانبية شديدة؟**
    a. قلق منخفض
    b. قلق شديد
    c. لا يوجد قلق
13. **هل تشعر بالقلق من أن تكشف نتائج اختبار الجينات المتعلق بالأدوية معلومات وراثية أخرى عنك، مثل احتمال الإصابة بأمراض معينة في المستقبل، حتى لو لم تكن هذه المعلومات مرتبطة بالدواء الذي تستخدمه؟**
    a. قلق منخفض
    b. قلق شديد
    c. لا يوجد قلق
14. **هل تشعر بالقلق من إمكانية وصول نتائج اختبارك الجيني الدوائي لأشخاص غير مختصين أو غير مصرح لهم؟**
    a. قلق منخفض
    b. قلق شديد
    c. لا يوجد قلق
15. **هل أنت قلق من أن تكون اختبارات الجينية المتعلقة بالأدوية مكلفة وغير مجدية؟**
    a. قلق منخفض
    b. قلق شديد
    c. لا يوجد قلق
16. **لدي مخاوف بشأن دقة وموثوقية الاختبارات الجينية المتعلقة بالأدوية.**
    a. قلق منخفض
    b. قلق شديد
    c. لا يوجد قلق

**الجزء ب – مخاوف بنعم/لا**

**خيارات الإجابة:**

- **نعم**
- **لا**

17. **في حال تم الكشف عن نتيجة سلبية هل تعتقد أنها قد تؤثر على وظيفتك أو الفرصة في الحصول على وظيفة؟**
    a. نعم
    b. لا
18. **في حال تم الكشف عن نتيجة اختبار غير مواتية، هل تعتقد أنك قد تكون في وضع غير مؤاتٍ أو تتعرض للتمييز من قِبَل شركات التأمين الصحي؟**
    a. نعم
    b. لا
19. **إذا كان عليك أن تدفع تكاليف الاختبارات الجينية الدوائية بنفسك، فإن التكاليف المالية ستكون أحد مخاوفك بشأن إجراء هذه الاختبارات.**
    a. نعم
    b. لا
20. **إذا كانت الاختبارات الجينية الدوائية مجانية أو تقوم شركة التأمين بتغطيتها، فسوف تكون أكثر استعداداً لإجرائها لتحسين اختيار الأدوية وتقليل المخاطر المرتبطة بالعلاج.**
    a. نعم
    b. لا

**الجزء ج – تفضيلات التواصل والموافقة**

**خيارات الإجابة:**

- موافق
- غير موافق

21. **من المهم أن يخبرني مقدم الرعاية الصحية عن هذه الاختبارات الجينية الدوائية، قبل إجراء أي منها وطلب موافقتي.**

- موافق
- غير موافق

22. **إذا كانت الإختبارات الجينية المتعلقة بالأدوية جزءًا من تحاليل الدم المعتادة، فمن المهم أن يسعى الطبيب المختص أو مقدم الرعاية الحصول على موافقة منفصلة مني، تحديدًا لإختبارات الصيدلة الجينية المتعلقة بالأدوية.**

- موافق
- غير موافق
